# Targeting cullin neddylation for cancer and fibrotic diseases

**DOI:** 10.7150/thno.78876

**Published:** 2023-09-04

**Authors:** Zhang-Xu He, Wei-guang Yang, Dan Zengyangzong, Ge Gao, Qian Zhang, Hong-Min Liu, Wen Zhao, Li-Ying Ma

**Affiliations:** 1 Pharmacy College, Henan University of Chinese Medicine, 450046, Zhengzhou, China.; 2 State Key Laboratory of Esophageal Cancer Prevention and Treatment; Key Laboratory of Advanced Pharmaceutical Technology, Ministry of Education of China; School of Pharmaceutical Sciences, Zhengzhou University, Zhengzhou, Henan 450001, China.; 3 Children's hospital affiliated of Zhengzhou university; Henan children's hospital; Zhengzhou children's hospital, Henan Zhengzhou 450000, China.; 4 China Meheco Topfond Pharmaceutical Co., Zhumadian 463000, China.; 5 Key Laboratory of Cardio-cerebrovascular Drug, Henan Province, Zhumadian 463000, China.

**Keywords:** Cullin neddylation, Inhibitors, Cancer, Fibrotic diseases

## Abstract

Protein neddylation is a post-translational modification, and its best recognized substrates are cullin family proteins, which are the core component of Cullin-RING ligases (CRLs). Given that most neddylation pathway proteins are overactivated in different cancers and fibrotic diseases, targeting neddylation becomes an emerging approach for the treatment of these diseases. To date, numerous neddylation inhibitors have been developed, of which MLN4924 has entered phase I/II/III clinical trials for cancer treatment, such as acute myeloid leukemia, melanoma, lymphoma and solid tumors. Here, we systematically describe the structures and biological functions of the critical enzymes in neddylation, highlight the medicinal chemistry advances in the development of neddylation inhibitors and propose the perspectives concerning targeting neddylation for cancer and fibrotic diseases.

## Introduction

**Figure 1:**
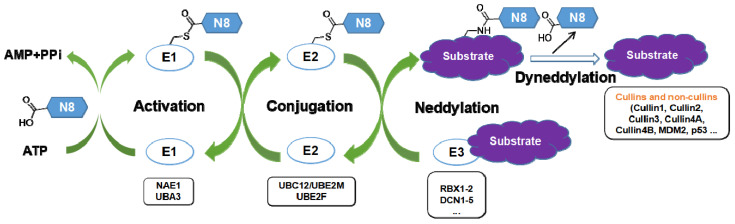
The three-step enzymatic reaction for protein neddylation. N8, NEDD8 (neural precursor cell expressed, developmentally down-regulated 8).

Neddylation, a reversible post-translational modification, adds a ubiquitin-like molecule NEDD8 (neuronal precursor cell-expressed developmentally downregulated protein 8) to a lysine residue of targeted substrate proteins through a three-step enzymatic cascade, catalyzed by NEDD8-activating enzyme E1 (NAE), NEDD8-conjugating enzyme E2s (UBC12/UBE2M or UBE2F) and substrate-specific NEDD8-E3 ligases (Figure 1) 1, 2. Shortly, the mature NEDD8 is firstly adenylated and activated in an ATP-dependent manner by NAE, a heterodimer comprising NAE1/APPBP1 and UBA3/NAEβ. Next, the activated NEDD8 is transmitted to one of two E2s, UBE2F or UBC12 *via* a trans-thiolation reaction, and then conjugated to its substrate protein through covalent attachment by various substrate-specific NEDD8 E3 ligases, such as RBX1/2 or DCN1-5 3, 4. Besides, neddylated substrates could be deneddylated by deneddylases, such as COP9 signalosome and NEDD8 protease 1 5-9. The best-identified physiological substrates of neddylation are cullin family proteins of CRLs, including cullin-1, -2, -3, -4A, -4B, -5, -7 and -9. UBC12 mainly interacts with RBX1 to mediate the neddylation of cullin-1, -2, -3, -4A and -4B, while UBE2F is highly selective to the neddylation of RBX2-related cullin-5 10-12. Cullin neddylation results in activation of CRLs, which are the biggest family of E3 ubiquitin ligases and control the ubiquitination of up to 20% cellular proteins for targeted degradation *via* the ubiquitin-proteasome system (UPS) 13-15. Accumulated evidence clearly demonstrates that many critical proteins (NAE, UBC12, UBE2F, DCN1, RBX1 and RBX2) in neddylation process are overactivated in multiple human diseases, such as cancers 16-22 and fibrotic diseases 23, 24. Furthermore, the abnormal expression of these enzymes is linked to poor patient prognosis 25, implying targeting neddylation as a promising therapeutic strategy for cancers and fibrotic diseases 26.

Currently, numerous efforts have been focused on developing novel agents for targeting cullin neddylation. MLN4924 (Pevonedistat), reported in 2009, is a first-in-class NAE inhibitor. It can effectively block neddylation of all cullins, causing the inactivation of CRLs and the accumulation of cancer-related substrates of CRLs 27-30. Preclinical studies have indicated that MLN4924 possesses robust antitumor activity, which is connected with cell cycle arrest 31, 32, apoptosis 30, 33, 34, senescence 35, 36, autophagy 37, inflammation 38 and angiogenesis 27, 39 in a cell-dependent manner. Given its impressive anticancer effect and well-tolerated toxicity in mice, MLN4924 is currently in many phase I/II/III clinical trials alone or in combination with chemotherapeutic drugs for cancer treatment. In addition to MLN4924, more and more neddylation inhibitors have been identified and show promising anticancer efficacy. Furthermore, the potential roles of neddylation inhibitors on fibrotic diseases are gradually being disclosed 23, 24.

Herein, we are intended to highlight numerous efforts on targeting cullin neddylation for cancer and fibrotic diseases. Specially, we summarize the structures and functions of critical neddylation proteins, the pivotal roles of neddylation in the progression of cancer and fibrotic disease as well as the advances in the development of neddylation inhibitors from a medicinal chemistry perspective. The opportunities and challenges of targeting cullin neddylation for cancer and fibrotic diseases are also discussed.

## Structures and functions of critical proteins in neddylation process

**Figure 2:**
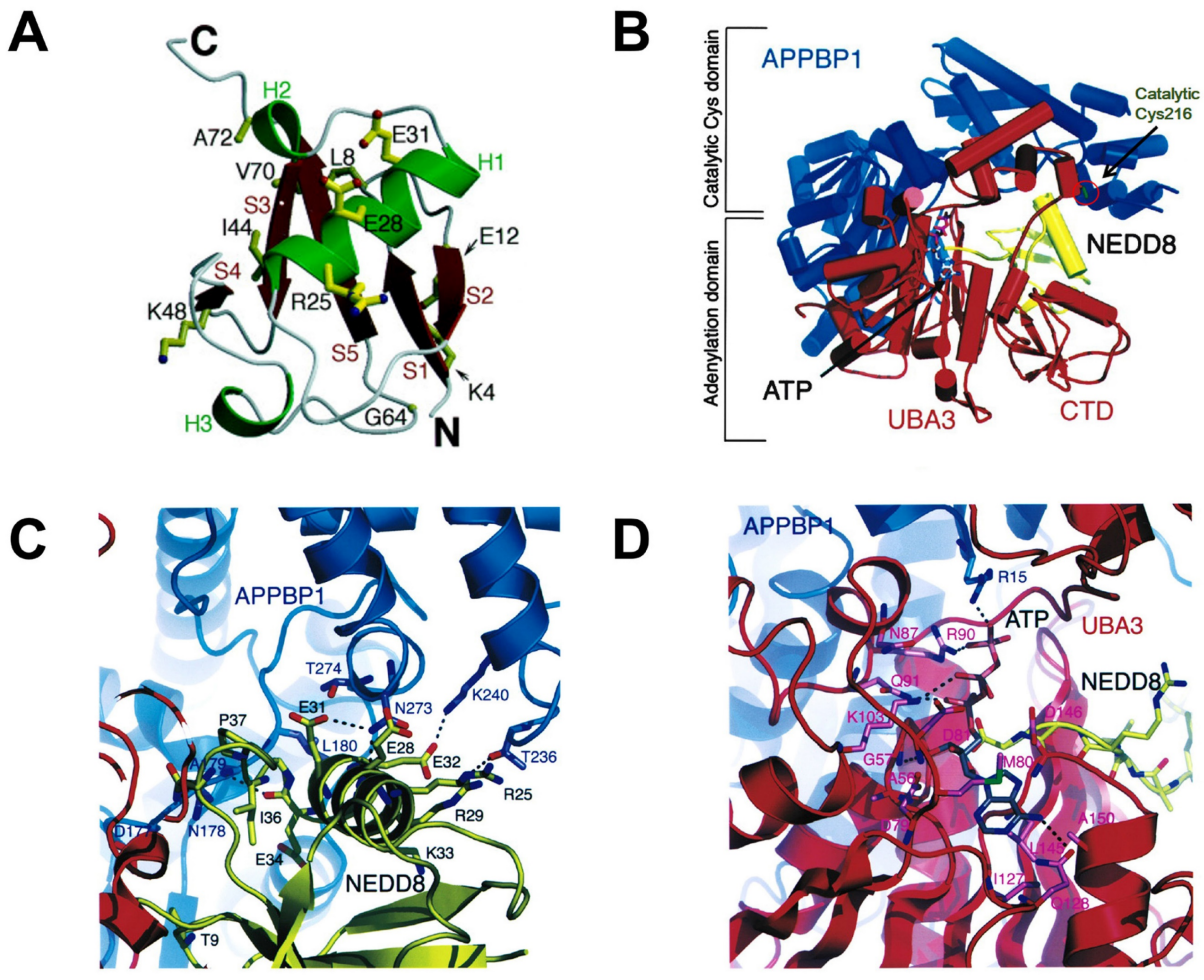
(A) The overall structure of NEDD8. Reproduced with permission 42. Copyright 1998, American Society for Biochemistry and Molecular Biology. (B) The structure of the APPBP1-UBA3-NEDD8-ATP complex. (C) The interactions between catalytic cysteine domain of NAE and NEDD8's acidic surface. (D) The interactions between NEDD8's C-terminal tail and ATP of adenylation domain. Reproduced with permission 43. Copyright 2003, Cell Press.

NEDD8, a polypeptide containing 81 amino acid residues, was cloned in mouse brain tissue in 1993. It possesses 80% similarity and 60% identical with ubiquitin and is overexpressed in the nucleus 40, 41. NEDD8 consists of a globular body containing a five-stranded mixed β sheet (red) and an α helix (green), a flexible C termini and N termini (Figure 2A). Additionally, NEDD8 has an asymmetric distribution of charged residues. One face of NEDD8 is primarily acidic and the other face comprises the Leu8/Ile44/Val70 hydrophobic patch 42, 43. The structure of APPBP1-UBA3-NEDD8 complex with ATP shows that NAE is composed of three domains: an adenylation domain having ATP binding site is connected *via* flexible loops to one domain at the C terminus of UBA3 subunit and another domain around the catalytic cysteine (Figure 2B). NEDD8 well fits in the groove of APPBP1-UBA3. The acidic face of NEDD8 in globular domain widely contacts with the catalytic cysteine domain of APPBP1 (Figure 2C). The hydrophobic patch of NEDD8 interacts with the adenylation domain, which is structurally conserved in the enzymatic cascade. Besides, NEDD8's C-terminal tail is inserted within 4A˚ of ATP's α-phosphate and ready for adenylation reaction (Figure 2D) 43, 44.

**Figure 3:**
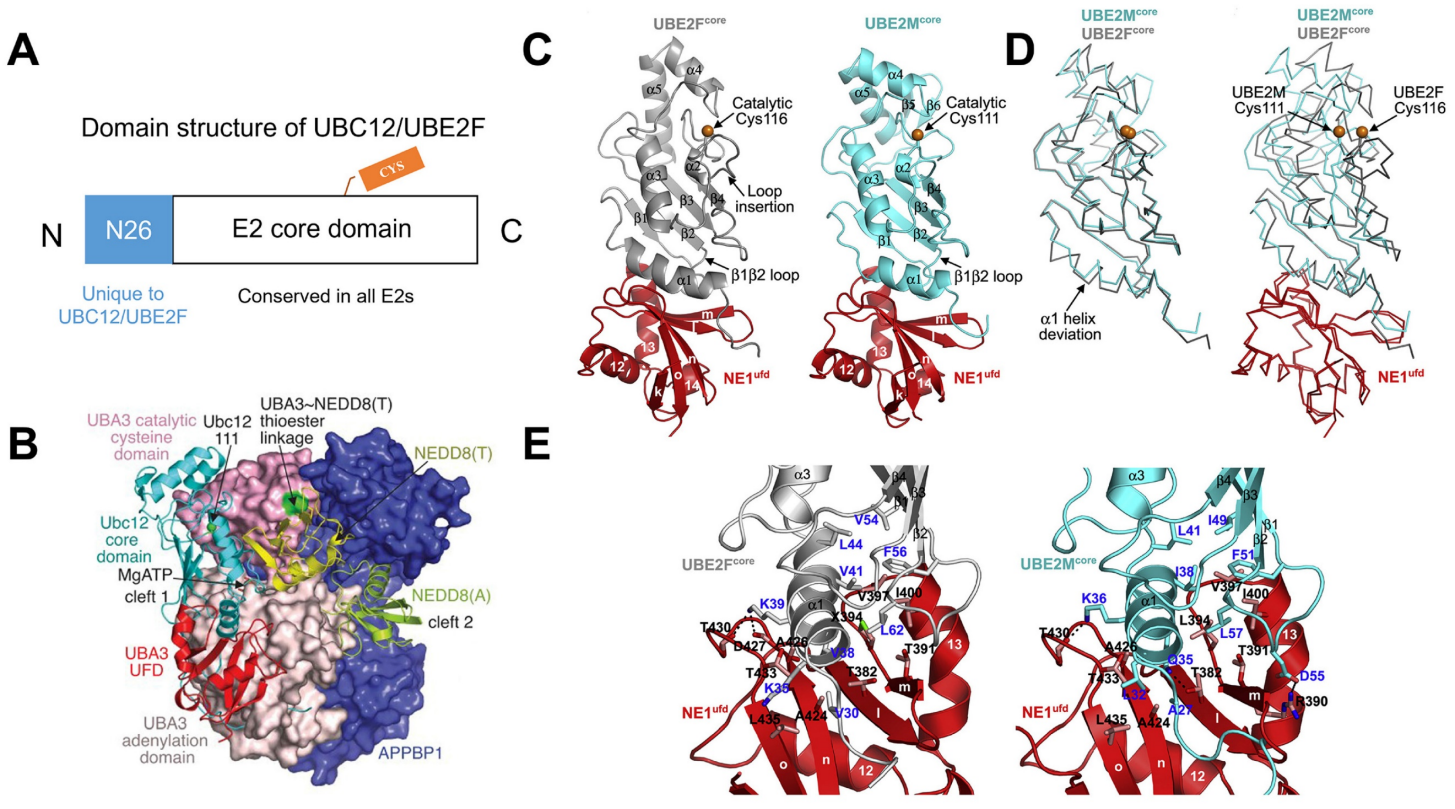
(A) The domain structure of UBC12/UBE2F. (B) Overall structure of the APPBP1-UBA3~NEDD8(T)-NEDD8(A)-MgATP-UBC12(C111A) complex. Reproduced with permission 47. Copyright 2007, Nature Publishing Group. (C) Overall structure of NAE^ufd^-UBE2F^core^ compared to NAE^ufd^-UBC12^core^. (D) The structural superposition of UBE2F^core^ and UBC12^core^. (E) The interactions between NAE^ufd^ and UBE2F^core^, NAE^ufd^ and UBC12^core^ separately. Reproduced with permission 12. Copyright 2009, Elsevier Inc.

The NEDD8 E2s (UBC12/UBE2M and UBE2F) generally include two regions: (i) a special 26-residue N-terminal extension identified only in UBC12/UBE2M and (ii) an E2 core region, a ∼150-residue domain conserved in all E2s, which possesses E2 catalytic cysteine (Figure 3A). The N-terminal domain can selectively recruit NAE to boost thioester formation between E2s and NEDD8 45. In the APPBP1-UBA3~NEDD8(T)-NEDD8(A)-MgATP-UBC12(C111A) complex, the three APPBP1-UBA3 domains pack to form a big groove, which links MgATP, two NEDD8 molecules and UBC12 together (Figure 3B). A crossover loop linking catalytic-cysteine and adenylation domains divides the groove into two parts. The peptide-like extension of UBC12 locates in a groove of UBA3's adenylation domain, and the core domain of UBC12 binds to UBA3's ubiquitin-fold domain (UFD). NE1^ufd^-UBE2F^core^ contains a globular ovoid structure like NE1^ufd^-UBE2M^core^, with UBE2F^core^ indicating a typical E2 catalytic core domain fold (Figure 3C). The superimposition of UBE2F^core^ and UBE2M^core^ shows different features, such as (i) different orientations of catalyzed cysteine, (ii) a half-turn extension and an offset orientation for UBE2F^core^'s N-terminal α1 helix, and (ⅲ) a prolonged C-terminal helix more reminiscent of ubiquitin E2 structures compared with two-stranded β sheet of C terminus in UBE2M (Figure 3D). Further structural analysis displays that NAE binds to different α helix and β1β2 loop sequences of UBE2F and UBE2M. The interactions are surrounded by two hydrophobic clusters between both E2s and NAE (Figure 3E). One cluster consists of NAE^ufd^'s Ala424, Ala426, Thr433 and Leu435, UBE2F^core^'s Val30 and the hydrophobic moiety of Lys35 from an α helix. These correspond to UBE2M^core^'s Ala27 and Leu32 separately. Another cluster comprises NAE^ufd^'s Thr382, Thr391, SeMet394, Val397 and Ile400 packing toward UBE2F^core^'s Val38, Val41 and Leu44 from an α helix; and Val54, Phe56 and Leu62 from the β1β2 loop. Even though Leu44, Phe56 and Leu62 are conserved between UBE2M^core^ and UBE2F^core^, other residues have apparent difference. For example, UBE2F^core^'s Val38 forms hydrophobic interactions, but the corresponding UBE2M^core^ Gln35 makes a hydrogen bond interaction with E1's Thr382. Also, NAE^ufd^-UBE2M^core^ complex is stabilized by many ionic interactions, whereas only one stabilizes NAE^ufd^-UBE2F^core^ (Figure 3E) 12, 46-48.

**Figure 4:**
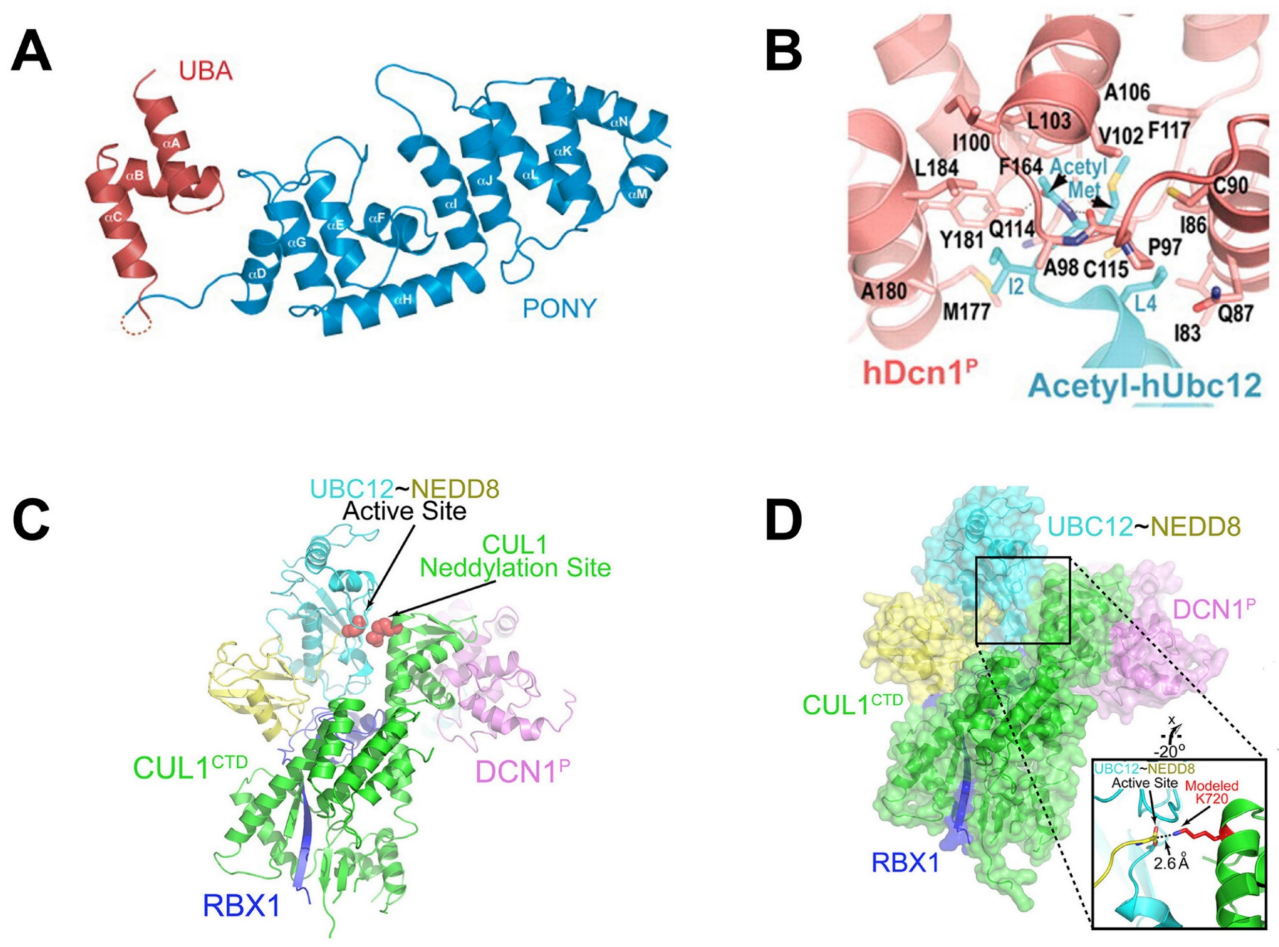
(A) Ribbon representation of full-length S. cerevisiae DCN1. The PONY and UBA domains are shown in blue and red separately. Reproduced with permission 50. Copyright 2008, Elsevier Inc. (B) Close-up of UBC12's acetylated N terminus binding to DCN1 in cartoon. Reproduced with permission 52. Copyright 2011, the American Association for the Advancement of Science. (C) Overall structure of RBX1-UBC12~NEDD8-CUL1-DCN1 complex as cartoon. (D) The surface of RBX1-UBC12~NEDD8-CUL1-DCN1 complex. Reproduced with permission 53. Copyright 2014, Elsevier Inc.

DCN-like proteins, as scaffold-like E3 ligases in neddylation process, do not need cysteine for catalytic function. They can interact with acetylated N-terminus of UBC12/UBE2F and cullin-RBX1 to increase neddylation efficacy 49, 50. DCN-like proteins include five isoforms (DCN1-5), each of which is composed of an N-terminal ubiquitin associated domain (UBA domain; residues 12-56), a disordered linker (residues 57-69) and a C-terminal domain (PONY domain; residues 70-269) (Figure 4A). Unlike the UBA domain, the PONY domain is mainly responsible for promoting neddylation. The N-terminal acetylation of NEDD8 E2s is usually vital for the activation of DCN-like proteins 49, 51. The structure of UBC12 complexed with DCN1 shows that interactions are mediated mainly by an N-terminal 12-residue peptide of UBC12 and a well-defined binding groove in DCN1 (Figure 4B) 52. Additionally, RBX1-UBC12~NEDD8-CUL1-DCN1 structure indicates that oxyester-bonded UBC12~NEDD8 intermediate is steady in complex with RBX1, DCN1^P^ and Cul1^CTD^ (Figure 4C-D). The center of the complex is the active site, in which Cul1 target site and UBC12~NEDD8 covalent linkage are juxtaposed within UBC12's catalytic center. The lysine's ε-amino group of a modelled Cul1 acceptor is ready for ligation at 2.6 A˚ from NEDD8's C-terminal carbon 53.

## Neddylation in cancers

Considering that neddylation proteins, including E1, E2s, E3s and neddylation substrates, are overactivated in various cancers and connected with cancer progression, targeting neddylation can be an appealing strategy for cancer treatment 11, 54, 55. The important roles of neddylation proteins in various cancers are summarized in the current section.

### The roles of neddylation in lung cancer

Lung cancer, one of the most common human malignancies, is regarded as a major public health concern around the world 56. Jia's group found that neddylation pathway was upregulated in both squamous-cell carcinoma and adenocarcinoma of lung, resulting in the increased neddylation of substrates, which induced degradation of tumor suppressors (such as p21 and p27) and promoted carcinogenesis. However, abrogation of neddylation markedly suppressed proliferation, migration, motility and survival of lung cancer cells. Similarly, treated with MLN4924 could also inhibit protein neddylation, inactivate CRL, lead to the accumulation of tumor-related CRL substrates and induce senescence or apoptosis 22. The treatment of lung cancer metastasis is still challenging, owing to lack of understanding of the potential mechanisms. In 2019, Jia's group recorded the metastatic process in real-time through a whole-mouse imaging system in Lewis lung carcinoma cells 57. The results showed that MLN4924 effectively blocked the metastatic process, including intravascular survival, extravasation and formation of metastatic colonies, thus inhibiting tumor metastasis. MLN4924 also suppressed the expression of matrix metalloproteinase 9 (MMP9), MMP2 and vimentin, interfered the actin cytoskeleton to weaken invasive potential and then resulted in a DNA damage response, cell cycle arrest as well as apoptosis. Tumor associated macrophages (TAMs) are the most abundant immune cell population infiltrating tumor microenvironment and are beneficial for tumor angiogenesis and metastasis. In addition, tumor-derived chemotactic cytokine ligand 2 (CCL2) is a monocyte-chemotactic protein. Its expression level is positively associated with numbers of TAMs in tumor tissues. Jia et al. discovered that the inactivation of neddylation pathway obviously inhibited the infiltration of TAMs, further suppressing lung cancer metastasis 58. Specifically, neddylation inactivation inhibited the activity of CRLs and led to the accumulation of its substrate IκBα to suppress nuclear factor-κB (NF-κB) transcriptional activity and CCL2 transactivation, thereby reducing TAMs infiltration.

Jia's group also observed that NEDD8 was upregulated in lung cancer, related to a worse patient overall survival 59. The knockdown of NEDD8 in lung adenocarcinoma exhibited significantly anticancer effects *in vitro* and *in vivo*. Further mechanism research revealed that NEDD8 downregulation caused the accumulation of some tumor-related CRLs substrates through inhibiting their degradation, thereby inducing cell G2 phase arrest, apoptosis and senescence.

Continually, Jia and co-workers indicated that the mRNA expression level of UBC12 in lung cancer tissues was much higher compared with that in normal lung tissues 60. Besides, the expression of UBC12 protein was not only positively related to the level of global protein neddylation, but also essential for maintaining the malignant phenotypes of lung cancer cells. Downregulation of UBC12 obviously decreased neddylation modification process, while overexpression of UBC12 enhanced protein neddylation. Importantly, UBC12 inhibition could effectively suppress lung cancer cell proliferation *in vitro* and *in vivo*. Meanwhile, UBC12 knockdown inhibited the growth of MLN4924-resistant cells by blocking cullin neddylation, leading to the accumulation of CRLs substrates. Mechanically, UBC12 knockdown blocked cullin neddylation, resulted in the inactivation of CRLs and caused the accumulation of tumor-related CRL substrates (p21 and p27) to further induce cell cycle arrest and inhibit the growth of lung cancer cells.

Sun and co-authors reported that UBE2F was upregulated in non-small cell lung cancer (NSCLC), and associated with poor patient survival 61. UBE2F overexpression contributed to lung cancer cell proliferation *in vitro* and *in vivo*, while its downregulation effectively inhibited tumor growth. Additionally, UBE2F coupled with RBX2 to induce Cul5 neddylation, resulting in the activation of CRL5 E3, which further enhanced the pro-apoptotic protein NOXA poly-ubiquitylation through atypical K11 linkage for proteasomal degradation. Thus, by promoting NOXA degradation, UBE2F could exert its growth-stimulating function. Platinum-based chemotherapy remains to be the main way against NSCLC, whereas the increasing occurrence of drug resistance has partly limited the clinical application of platinum-based chemotherapy. Jia's group found that the upregulation of UBE2F was a critical approach for lung cancer cells to escape platinum-induced cell apoptosis, causing insensitivity to platinum-based chemotherapy 62. Deletion of UBE2F could sensitize lung cancer cells to platinum treatment by increasing the protein levels of pro-apoptotic protein NOXA and subsequently inducing cell apoptosis. Mechanistically, platinum treatment damaged the interaction of UBE2F-RBX1 complex and thus suppressed its degradation. Additionally, the accumulated UBE2F stimulated the neddylation levels and activity of Cul5, in accord with the decreased expression of NOXA, a well-known substrate of CRL5.

The neddylation E2 conjugating enzyme consists of UBC12 and UBE2F, while whether and how both E2s cross-talk with each other is unexplored. Sun and co-workers proposed that UBC12 was a stress-inducible protein, exposed to upregulation by AP-1 and HIF-1, whereas UBE2F was degraded by UBC12-associated E3 ligases. UBC12 served as a neddylation E2 to activate CRL3 Keap1 to maintain UBE2F level upon normal physiological conditions. Upon hypoxia or mitogen stimulation, UBC12 acted as a dual E2 complexed with DJ-1/Parkin to enhance ubiquitylation and degradation of UBE2F, further resulting in CRL5 inactivation, the pro-apoptotic protein NOXA accumulation and the growth inhibition of lung cancer cells 63.

Defective in cullin neddylation 1 (DCN1) is also known as DCUN1D1, DCNL1 or squamous cell carcinoma-related oncogene (SCCRO). It serves as a co-E3 together with RBX1 to promote transfer of NEDD8 from its E2 (UBC12) to cullins, further regulating CRL stability 20. DCN1 is connected with tumor development and poor outcomes in NSCLC and squamous cell carcinoma (SCC) 64, 65. Also, DCN1 expression is closely related to tumor stage in patients with NSCLC. It was found in none of the patients with stage I disease, 10% of those with stage II disease and 29% with stage III disease. Moreover, 14 of 16 DCN1 positive patients led to brain metastasis. Besides, knockdown of DCN1 inhibited the growth of 3q26-29-amplified SCC cells 66. Aberrant expression of circular RNAs (circRNAs) is linked to cancer progression. Liang and co-authors screened circRNA expression of A549 cells, identifying circDCN4, which could inhibit glycolysis and metastasis 67. Besides, the downregulation of circDCN4 was more common in lymph node metastatic tissues and acted as an important risk factor for the whole survival of lung adenocarcinoma patients.

Cul1 is the scaffold component of SCF (Skp1-Cul1-F-box protein) complexes that control the proteolysis of many proteins associated with cell cycle progression. The abnormal expressions of F-box proteins were found in human cancers, such as lung cancer. Salon et al. reported that the upregulation of Cul1 was observed in 40% (51/128) of all lung tumors in comparison to normal lung tissues, including 75% (18/24), 30% (16/54) and 34% (17/50) of carcinoids, high grade neuroendocrine lung carcinomas and NSCLCs separately 68. Additionally, high levels of Cul1 protein could protect cells from hyperproliferative signals through cyclin E downregulation. Moreover, neddylated forms of Cul1 were highly expressed in neuroendocrine lung tumors. These results show that alterations of Cul1 neddylation can promote the development of highly aggressive lung tumors.

### The roles of neddylation in liver cancer

Liver cancer includes hepatocellular carcinoma (HCC), bile duct cystadenocarcinoma, intrahepatic cholangiocarcinoma (ICC), epitheliod haemangioendothelioma, hepatoblastoma and haemangiosarcoma. In particular, HCC has relatively heterogeneous pathogenesis making it challenging for the existing therapies 69. The overactivation of neddylation pathway was found in HCC patients with a positive correlation between overall levels of neddylation and poorer prognosis, suggesting that neddylation modification may be a promising therapeutic approach for HCC clinical management 70, 71. Jia and co-workers disclosed that the effect of MLN4924 on inducing autophagy was related to the CRL inactivation, which was vital for inhibiting the proliferation of liver cancer cells. In addition, MLN4924-induced autophagy was partly linked to inhibition of the mammalian target of rapamycin (mTOR) activity and induction of reactive oxygen species (ROS) stress. Besides, inhibiting autophagy promoted MLN4924-induced apoptosis 72. Subsequently, Jia and co-authors also examined the clinical relationship and therapeutic potency of targeting neddylation pathway in ICC through analyzing the immunohistochemistry of neddylation proteins in a cohort of 322 cases 16. The results presented that E1, E2s and global NEDD8 conjugation were overactivated in over 2/3 of human ICC. Besides, MLN4924 treatment obviously suppressed cholangiocarcinoma cell proliferation and tumor growth with synergistic effect when combined with cisplatin.

Jia's group also found that tumor suppressor RhoB was a new substrate of the neddylation-CRL pathway. Ablation of RhoB could decrease the expression of tumor suppressor p21 and p27. Cul2-RBX1 E3 ligase, as the main regulator, functioned for the ubiquitination and degradation of RhoB in liver cancer cells. The overactivation of neddylation-CRL pathway enhanced the degradation of RhoB, thus promoting liver carcinogenesis and tumor progression. However, treated with MLN4924 blocked cullin neddylation, inactivated CRL and caused the accumulation of RhoB, which further induced apoptosis and suppressed proliferation of liver cancer cells 73.

Liver metastasis is the main reason of death in patients with uveal melanoma (UM), thus it is urgently required to develop an effective therapy. A recent study has shown that MLN4924 could suppress cancer stem-like cell properties in UM *via* Slug protein degradation. Additionally, MLN4924 interfered the secretion of NF-kB-mediated VEGF-C and angiogenesis. Besides, neddylation inhibition on proliferation was associated with G2/M phase arrest and activation of ATM-Chk1-Cdc25C DNA damage response. These findings show that neddylation inhibition is beneficial for the treatment of hepatic metastasis of UM 74.

Recently, the potential roles of UBC12 in ICC have been explored by Zhao et al 75. They found that ICC patients with overexpressed UBC12 showed worse accumulative recurrence and overall survival in comparison to patients with low expression. However, the knockdown of UBC12 caused the decreased viability and colony formation of ICC cell lines, possibly owing to DNA damage responses and apoptosis. Besides, UBC12 silencing inhibited ICC tumor growth, as demonstrated by the decreased size of tumor xenografts infected with the shUBC12 lentivirus. Although knockdown of UBC12 with siRNA can inhibit neddylation pathway and lead to a robust anticancer effect, how to transport siRNA with low toxicity *in vivo* remains to be solved. Li's group designed a folic acid-modified (poly(lactic-co-glycolic acid))-(thioketal)-(polyethylene glycol) nanomedicine for siRNA delivery 76. The nanomedicine not only suppressed proliferation and induced apoptosis of liver cancer cells, but also showed favorable therapeutic ability through silencing UBC12, inhibiting neddylation pathway and accumulating tumor-suppressive CRL substrates with low toxicity. More importantly, the nanomedicine exhibited promising potency for multifunctional nano-system applicable in cancer therapy.

RING box protein-1 (RBX1), also named ROC1, is an important RING component of CRL and overactivated in hepatocellular carcinomas connected with poor prognosis. Jia and co-workers found that knockdown of RBX1 suppressed the proliferation of liver cancer cells *via* inducing autophagy and p21-dependent senescence. Further exploration displayed that RBX1 silencing activated autophagy through suppressing mTOR activity, owing to the accumulation of mTOR-inhibitory protein Deptor, a substrate of CRL. Mechanically, autophagy response on RBX1 silencing was a survival indicator. Additionally, the inhibition of autophagy pathway sensitized cancer cells to apoptosis. The above results not only disclosed RBX1 as an attractive drug target for liver cancer, but also provided a new chance for liver cancer treatment through combining RBX1 inhibitor with autophagy inhibitor 77.

Cul1 acts as a vital scaffold of Skp1-Cul1-F-box protein (SCF) E3 ubiquitin ligase complex that regulates ubiquitination of proteins related to cell cycle, transcription and signal transduction. To explore the role of Cul1 in HCC, Liu et al. determined Cul1 expression by immunohistochemistry using 90 cases HCC tissues and the corresponding adjacent non-cancerous tissues. The results indicated that in comparison to the paired adjacent non-tumor tissues, Cul1 expression was evidently increased in HCC tissues. Also, Cul1 staining was apparently connected with tumor size, TNM stage and histology grade. Besides, the overexpression of Cul1 was a strong independent prognostic indicator of HCC and closely correlated with worse 5-year overall rates in HCC patients. These data demonstrated that Cul1 could be a key prognosis marker for human HCC 78.

Cul4A is also a scaffold protein for E3 ubiquitin ligase and overexpressed in HCC tissues. A negative correlation was observed between Cul4A expression and tumor differentiation grade as well as patient survival, but a positive relationship with venous invasion, lymphatic and hepatocyte proliferation. Additionally, Cul4A knockdown promoted the expression of epithelial marker (E-cadherin), but reduced the steady-state level of mesenchymal marker (Vimentin) as well as EMT-related transcription factors, indicating that Cul4A could promote the development of HCC by stimulating EMT trend. Moreover, Cul4A downregulation suppressed the growth of HCC cells, resulting in the reduction of S-phase and inhibition of Cyclin A and Cyclin B1. The above findings show that Cul4A may be a promising target for HCC treatment 79.

Similarly, Cul4B displays tumor-promoting effects and is noticeably upregulated in different cancers. Yuan et al. observed that overexpression of Cul4B enhanced spontaneous and DEN-induced hepatocarcinogenesis. Additionally, the increased hepatocarcinogenesis in Cul4B transgenic mice could be regulated by the increased proliferation and oxidative stress. Besides, the transgenic mice revealed stronger compensatory proliferation after DEN-induced liver injury, accompanied by the activation of Erk, NF-κB, p38 and Akt. Taken together, Cul4B plays a critical role in hepatocarcinogenesis 69.

Cul7, a non-typical cullin protein comprising approximately 1,700 amino acids, serves as a component of the SCF complex and interacts with RBX1, Fbw8 and Skp1. Zhang et al. found that Cul7 was overexpressed in HCC tumor tissues, particularly in metastatic HCC tumor tissues. In addition, there was an adverse relationship between Cul7 expression and extended survival. Silencing of Cul7 could effectively inhibit invasion, migration and metastatic capacities of liver cancer cells. Besides, upregulation of Cul7 enhanced epithelial-mesenchymal transformation, demonstrated by the increased mesenchymal cell markers (N-cadherin, vimentin and fibronection) and reduced epithelial cell markers (E-cadherin and a-catenin) 80.

### The roles of neddylation in leukemia

Acute myelogenous leukemia (AML) is characterized by maturation arrest and hyperproliferation of clonal myeloid precursors. Chemotherapy partly meets the clinical application, whereas patients with AML may relapse because of the occurrence of drug resistance with increased cancer cell ability to rescue DNA damage. Therefore, new therapies are urgently needed 81. Swords et al. reported that MLN4924 could effectively induce AML cell death and stabilize important NAE targets, further suppressing NF-κB activity, DNA damage and ROS generation. Besides, administration of MLN4924 significantly suppressed AML xenografts growth 33. Overexpression of microRNA-155 (miR-155) is connected with poor survival in AML. The miR-155 is controlled by NF-κB, the activity of which is partly regulated by the NEDD8-related ubiquitin ligases. Marcucci's group revealed that MLN4924 reduced the binding of NF-κB to miR-155 promoter and downregulated miR-155. Also, overactivation of miR-155 decreased MLN4924-induced antileukemic potency. Moreover, MLN4924 could induce miR-155 expression and prolong the survival of mice xenografted with human leukemic cells 82.

Acute promyelocytic leukemia (APL) is characterized by the arrest of terminal differentiation of myeloid cells into granulocytes. All-trans retinoic acid (ATRA) treatment can clinically relieve APL through inducing granulocytic differentiation and then cell death of differentiated leukemic cells. Wang and co-authors reported that neddylation pathway was involved in the treatment of ATRA for APL, and ATRA treatment in primary APL cells resulted in the degradation of UBA3. Moreover, neddylation inhibition was able to induce differentiation, apoptosis and proliferation suppression 83. Meanwhile, Liu et al. found that ATRA could decline the expression of NAE, further suppressing neddylation of Cul1 and Cul3 in the APL cell line NB4 84. Also, MLN4924 suppressed proliferation and induced apoptosis of APL cells *via* inhibiting cullin neddylation. Importantly, MLN4924 could promote ATRA-induced differentiation of APL cells by inducing autophagy.

Chronic myeloid leukemia (CML) is involved in the fusion of Abelson oncogene (ABL) with breakpoint cluster region (BCR) gene to encode BCR-ABL, which is the only driving force in pathogenesis of CML. Both resistance to imatinib owing to T315I mutation and leukemia stem cells (LSC) are challenging in patients with CML. Pan and co-workers reported that blocking of neddylation suppressed BCR-ABL point mutations and LSCs, which were responsible for imatinib-resistant recurrences. Biologically, they verified that MLN4924 could prompt CML cell G2/M phase arrest and apoptosis. Besides, MLN4924 inhibited the growth of human CML and LSC cells in CML-bearing mice. These findings show neddylation inhibition as a promising therapeutic strategy for CML with LSC-derived imatinib resistance 85.

Chronic lymphocytic leukemia (CLL) is characterized by the accumulation of neoplastic B cells, resistant to apoptosis. Constitutive and BCR-dependent activation of NF-kB is a vital feature in CLL B cells and forecasts poor survival, but its activation may result in tumor resistance. Therefore, neutralization of NF-kB is an attractive strategy for CLL, which not only has the potential to inhibit the proliferation of CLL cells, but also leads to sensitization to both BCR-targeting agents and conventional chemotherapy. Interestingly, NEDD8 can regulate the activity of CRLs, therefore indirectly controlling NF-κB activity. Danilov's group found that MLN4924 disrupted NF-kB activation and caused Bim expression in CLL cells, further blocking stroma-mediated resistance 86. Similarly, Godbersen et al. reported that both pulse and continuous exposure to MLN4924 could abolish NF-κB activity and induce apoptosis in CLL B cells, thus circumventing stroma-mediated resistance 87. NF-κB pathway activity is essential in immune response. In addition, T-cell function is changed in patients with CLL. Danilov's group indicated that MLN4924 abrogated NF-κB signalling in malignant B cells 88. Besides, T cells treated with MLN4924 recovered NAE activity, and maintained their response to T-cell receptor stimulation and cytotoxic potential, suggesting the immune role of targeting neddylation in lymphoid malignancies and CLL.

### The roles of neddylation in glioblastoma

Glioblastoma (GBM) is the most malignant primary brain tumor, with limited treatment options. Jia's group firstly reported that neddylation pathway was overactivated in GBM tumor tissues in comparison to adjacent normal tissues 18, 89. The upregulation of neddylation pathway was positively related to postoperative recurrence and high-grade disease, while was adversely connected with overall survival. Moreover, treated with MLN4924 significantly suppressed cullin neddylation and inactivated CRL, further inducing senescence, G2 cell cycle arrest and apoptosis. More importantly, inhibition of neddylation by MLN4924 inhibited tumor growth in a xenograft model of human GBM without obvious side effect. Further exploration showed that recurrent GBMs possessed higher neddylation activity than primary GBM 90. Continually, Han et al. found that NEDD8 E1 (NAE) levels were increased in glioblastoma patients and high NEDD8 levels were connected with poor clinical outcomes. In addition, both patient-derived stem cells and glioblastoma cells were very sensitive to MLN4924, whereas healthy human astrocytes were resistant. Interestingly, MLN4924 could inhibit ERK and AKT phosphorylation in MLN4924 sensitive cells 91.

Programmed death ligand-1 (PDL1) is a central immunological checkpoint ligand molecule, and its overexpression in gliomas leads to the shortening of patient survival and reduction of the antitumor immune response. Filippova and co-workers reported that MLN4924 showed strongly antiproliferative activity against PDGx and glioma cell lines with the IC_50_ values ranging from 0.2 to 3 uM. Additionally, MLN4924 treatment increased the expression of PDL1 and its main transcriptional enhancer hypoxia-inducible factor 1A (HIF1A) in all glioma cell lines, which might cause potential resistance through evasion of immune surveillance checkpoints. Moreover, the overexpression of PDL1 in glioma cells caused T-cell energy, which could be inhibited by PD1/PDL1 blockage 92. Continually, Wang's group reported that silencing of neddylation pathway key enzymes strikingly enhanced PDL1 expression in glioblastoma cancer cells. MLN4924 could increase PDL1 mRNA levels by suppressing WD repeat domain-containing 7 E3 ligase activity and Cul1-F-box, and accumulating c-MYC. Additionally, MLN4924 reduced T cell killing *via* PDL1 induction. However, the inhibition of PDL1 recovered sensitivity of MLN4924-treated glioblastoma cancer cells to T cell killing. To sum up, the combination of PD1/PDL1 inhibition and MLN4924 may be an effective strategy for glioma treatment 93.

Zheng et al. indicated that overexpression of CRL5 components SOCS3, Elongin C, Elongin B and RBX2 forecasted poor prognosis of glioma and all GBM subclasses. In addition, the high protein levels of Cul5 were negatively connected with those of p65/RelA and vHL. Moreover, GBM neovascularization could be enhanced by CRL5-mediated anti-angiogenic vHL protein downregulation. This study shows that CRL5 inhibition may relieve GBM progression 94. Positive epidermal growth factor receptor (EGFR) immunoreactivity in GBM is used to forecast poor radiation response. Unfortunately, numerous efforts to target EGFR have not been successful because of the molecular heterogeneity of EGFR expression in GBM. Considering that CRL2 can degrade EGFR, Cyclin B1 and HIF-1a, it is valuable to explore the probable role of CRL2 on GBM survival and radiosensitivity. Zheng et al. discovered that expression of Cul2 could forecast GBM development and survival rate. Cul2 protein levels were negatively corrected with those of Cyclin B1, EGFR, HIF-1a and VEGF-A, suggesting that patients with high Cul2 expression level may not develop resistance toward EGFR inhibitors. Besides, elevated Cul2 expression predicted increased radiosensitivity. Together, Cul2 can be regarded as a biomarker in facilitating GBM and radiosensitivity profiling 95.

### The roles of neddylation in head and neck squamous cell carcinoma

Head and neck squamous cell carcinoma (HNSCC) arises in the nasal cavity, larynx, pharynx and oral cavity, and shows poor survival rates in advanced stages. Jia's group reported that the expressions of NAE and UBC12 were upregulated in HNSCC tissues than that in normal tissues. Additionally, neddylation inhibition evidently suppressed the abnormal proliferation of HNSCC cells. Mechanistically, MLN4924 could cause the accumulation of CRL ligase substrate c‐Myc that transcriptionally activated pro‐apoptotic protein Noxa, finally resulting in apoptosis of HNSCC 96.

CRL4 with its substrate receptor CDT2 (CRL4-CDT2) is an important regulator of DNA replication *via* targeting CDT1, p21 and SET8 for ubiquitin-dependent proteolysis. The abnormal enhanced stability of these proteins can lead to DNA re-replication in cells with inactivated CRL4-CDT2, which is harmful to cells. Abbas and co-authors uncovered that CDT2 was upregulated in HNSCC. Its silencing significantly inhibited the growth of human papilloma virus negative (HPV-ve) HNSCC cells mainly *via* the induction of re-replication. In addition, MLN4924 treatment could induce re-replication, inhibit HNSCC cell proliferation as well as HNSCC xenografts growth. Besides, MLN4924 not only sensitized HNSCC cells to ionizing radiation (IR), but also increased IR-induced inhibition of xenografts in mice. These findings display that induction of re-replication is a new manner to treat radioresistant HNSCC tumors. MLN4924 may act as an adjuvant for IR-based treatments 97.

Chairatvit et al. found that NEDD8 conjugation was upregulated in human oral carcinoma cells. The expression of NEDD8 conjugation was mainly related to the concentration of serum, which also influenced the proliferation of oral squamous cell carcinoma. Importantly, NEDD8 conjugation was necessary for the proliferation of carcinoma cell as the transfection of negative UBC12 could increase proliferation rate 98.

Nasopharyngeal carcinoma (NPC) is one of the most common human malignancies in south China, with a high recurrence rate and treatment resistance. Qian's group reported that both knockdown of NEDD8 by shRNA and MLN4924 treatment significantly inhibited NPC cell proliferation, apoptosis and cycle arrest, whereas overexpression of NEDD8 displayed counter effects. Additionally, the inactivation of NEDD8 increased the sensitivity of NPC cells to cisplatin and radiation. These results reveal that NEDD8 is a novel prognostic marker and MLN4924 may act as an appealing therapeutic target for NPC treatment 21.

14‐3‐3ζ is known as a potential oncogene related to the pathogenesis of HNSCC. Seo and co-workers indicated that knockdown of 14‐3‐3ζ dramatically resulted in senescence phenotypes through p27 accumulations. Cul1 neddylation was reduced *via* 14‐3‐3ζ silencing. Additionally, 14‐3‐3ζ silencing suppressed the growth of Hep‐2 cells implanted into nude mice. However, downregulation of CSN5, a deneddylase, recovered the senescence induced by 14‐3‐3ζ depletion. In summary, 14‐3‐3ζ silencing caused premature senescence in Hep‐2 laryngeal cancer cells, accompanied by upregulation of p27, which might be correlated with the inactivation of the SCF ubiquitin ligase *via* the deneddylation of Cul1 99.

### The roles of neddylation in melanoma

Melanoma arises from the melanocyte lineage and is the most lethal form of skin cancer, including four anatomic subtypes (mucosal, cutaneous, uveal, and acral) 100. Sun's group reported that NAE was upregulated in melanoma cell lines and tissues. The silencing of UBA3, a subunit of NAE, significantly inhibited the proliferation of melanoma M14 cells, led to G0/G1 arrest and apoptosis, suggesting UBA3 as a potential target for melanoma 101, 102. CDT2 was also overactivated in melanoma. The knockdown of CDT2 could inhibit the proliferation of melanoma cells through a mechanism that was dependent on the stabilization of CRL4^CDT2^ substrates SET8 and p21. Stimulatingly, MLN4924 revealed strongly anti-melanoma activity by inducing SET8- and p21-dependent senescence and re-replication. These results showed that CRL4-CDT2-SET8/p21 degradation axis could be the main target of MLN4924 in melanoma 103. Similarly, Wood and co-workers discovered that MLN4924 dramatically inhibited the viability of canine melanoma cells, and induced apoptosis by induction of DNA re-replication and cell senescence 104. Moreover, most canine melanoma samples displayed sensitivity to MLN4924 of nanomolar concentration, which was related to P21 levels. Continually, Wong et al. evaluated the anticancer efficacy of MLN4924 using melanoma cells and patient-derived tumor xenografts (PDTX). The results indicated that MLN4924 could suppress cell growth (IC_50_ < 0.3 μM) and induce melanoma cell apoptosis. Also, MLN4924 inhibited tumor growth in melanoma cell xenografts and PDTX 105. These results presented neddylation inhibition as a promising strategy for melanoma treatment.

### The roles of neddylation in colorectal cancer

Colorectal cancer with high morbidity and mortality, is one of the most frequently diagnosed cancers in the world 106. Gao's group reported that MLN4924 not only suppressed colon cancer cell (HCT116 and HT29) growth, but also induced cell G2/M phase arrest and apoptosis. Additionally, MLN4924 significantly triggered autophagy in HCT116 and HT29 cells through inhibiting PI3K/AKT/mTOR pathway, whereas treated with the autophagy inhibitor 3-MA blocked the effect of MLN4924 on inhibiting colon cancer cell growth and cell death. Moreover, MLN4924 effectively inhibited colon cell proliferation in a xenograft model 107. Fotouhi and co-authors indicated that both NEDD8 and RBX1 were overexpressed in small intestinal neuroendocrine tumors (SI-NETs) from patients with liver metastasis. MLN4924 treatment displayed potent antiproliferative activity and induced SI-NET cell apoptosis. Besides, proteomics analysis upon neddylation inhibition in two SI-NET cells led to the upregulation of CRL substrates. Furthermore, the inactivation of CRL, by either NEDD8/RBX1 silencing or MLN4924 treatment induced SI-NET cell apoptosis, which was partly rescued by silencing of p27 108. NOXA, a BH3-only proapoptotic protein, is overexpressed in colorectal cancer (CRC). Zhang's group found that PRDX1, an antioxidant overexpressed in CRC, could suppress CRC cell apoptosis by downregulating NOXA. Mechanistically, PRDX1 caused NOXA ubiquitination and degradation, which absolutely depended on Cul5 neddylation. Additionally, PRDX1 oligomers could bind to both UBE2F and Cul5, and this tricomplex was important for Cul5 neddylation, as downregulation of PRDX1 could suppress Cul5 neddylation and NOXA degradation 109.

### The roles of neddylation in prostate cancer

Prostate cancer (PCa), as the third primary cause of death in men with cancer, is the most common non-cutaneous malignancy. In 2002, Meehan et al. found that the expression of NEDD8 was downregulated in prostate cancer tissues in comparison to normal prostate tissues using proteomics 110. Subsequently, Guo's group reported that neddylation pathway was critical and targetable in PCa. MLN4924 treatment not only inhibited PCa cell proliferation and clone formation, but also caused DNA damage, G2 phase arrest and apoptosis, which was related to the inactivated CRLs, accompanied by accumulation of tumor-suppressive CRLs substrates 111. Continually, Rulina and co-workers probed CRLs in PCa cells and found significant plasticity of cells with TMPRSS2-ERG translocation. In addition, CRL suppression by knockdown of RBX1 or chemical inhibition resulted in prostate cancer cell G0/G1 phase arrest that inhibited cell apoptosis. Besides, inhibition of neddylation-dissociated 1 protein (Cand1), cullin-associated targets and androgen receptor (AR) sensitized cancer cells to CRL inhibition 112. Recently, Balakirev's group analyzed the effect of MLN4924 on spheroids formed by prostate cancer LNCaP and VCaP cells, and found that MLN4924 had diverse effects on the tested cell lines, differing from cell cycle arrest and protective dormancy to apoptosis and senescence 113. AR and its active variants (AR-Vs) are closely connected with the development and recurrence of PCa. In 2020, Sun's group indicated that both MLN4924 treatment and knockdown of NAE effectively inhibited growth and invasion of PCa cells without significant effects on normal prostate epithelial cells. Additionally, the combination of MLN4924 with castration or AR antagonist displayed more potent anti-prostate cancer activity *in vitro* and *in vivo*, compared with monotherapy. Biologically, MLN4924 suppressed the transcription of AR/AR-V7 and its downstream targets, and the expressions of MMP2 and MMP9 114.

### The roles of neddylation in breast cancer

Recently, neddylation inhibition has been reported as an appealing way for breast cancer treatment 115. Jia and co-authors indicated that neddylation was overactivated in ER-positive breast cancer and forecasted poor prognosis. Treated with MLN4924 downregulated ERα expression* in vitro* and *in vivo*. Additionally, MLN4924 single or synergized with fulvestrant dramatically inhibited the growth of ER-positive breast cancer 116. The overexpression of estrogen-related receptor beta (ERRβ) is positively associated with the improved prognosis and prolonged survival in breast cancer patients. Mishra's group presented that ERRβ was the main substrate of SCF complex. In breast cancer, neddylation could trigger cullin subunits of SCF complex to target ERRβ for further degradation. However, MLN4924 treatment restored ERRβ expression and led to decreased proliferation and migration of breast cancer cells. Besides, the role of ERRβ was not related to the expression of Erα 117.

### The roles of neddylation in esophageal cancer

Esophageal squamous cell carcinoma (ESCC) is a primary type of esophageal cancers ranked as the sixth main cause of cancer death worldwide 118. Jia's group reported that neddylation pathway was overactivated in ESCC and adversely connected with patient survival. Inhibition of neddylation pathway could induce death receptor 5 (DR5)-mediated apoptosis and cause the inhibition of ESCC *in vivo*. Further studies showed that neddylation inhibition stabilized CRL substrate activating transcription factor 4 (ATF4), which transactivated transcription factor CHOP, resulting in the expression of DR5 to activate caspase-8 and induce extrinsic apoptosis 119. Continually, Jia and co-workers found that MLN4924-induced neddylation inhibition caused the accumulation of ATF3 to promote prosurvival autophagy by regulating NF-κB-Catalase-ROSATF3 axis in esophageal cancer cells, suggesting inhibition of ATF3-mediated autophagy as a promising strategy to improve neddylation mediated ESCC treatment 120. Recently, Augoff's group also exhibited that MLN4924 possessed a significantly inhibitory efficacy on the tumor necrosis factor-alpha (TNF-α)-induced activity of MMP9 in ESCC cells, which further inhibited MMP9-dependent cancer cell migration 121. Wang et al. discovered that UBC12 was upregulated in ESCC tissues and forecasted a poor survival of patients. Knockdown of UBC12 could trigger the tumor-suppressive cellular responses of ESCC cells, and suppress malignant phenotypes of ESCC cells. Besides, downregulation of UBC12 inhibited CRLs and caused the accumulation of CRLs substrates (e.g. p21 and p27), consequently prompting DNA damage, cell cycle arrest, apoptosis or senescence 122.

### The roles of neddylation in gastric cancer

Neddylation inhibition is regarded as a promising strategy for gastric cancer (GC) treatment 123. Zhang and co-authors reported that MLN4924 possessed robust antiproliferative activity against GC cells, accompanied by the accumulation of tumor-suppressor gene P27 and G2/M phase arrest. P27 inactivation in MLN4924-treated cells led to more potent inhibitory activity *in vitro* and *in vivo*, which was related to the overproduction of ROS. Additionally, P27 knockdown influenced the expression levels of BH3 family members to enhance the mitochondrial dysfunction and apoptosis. These findings not only uncovered the protective role of P27 in MLN4924-treated GC cells, but also implied that inhibition of MLN4924 with P27 might generate synergistic therapeutic efficacy 124. Sun's group also found that MLN4924 could suppress Cul1 neddylation, along with the inhibitory proliferation and migration in GC cells. Mechanically, MLN4924 caused the accumulation of numerous CRL substrates to promote DNA damage and induce G2/M phase arrest, senescence as well as autophagy. Besides, MLN4924 could also depressed gastric cancer cell migration by transcriptionally activating E-cadherin and inhibiting MMP-9 125.

### The roles of neddylation in myeloma

In 2012, McMillin et al. reported that MLN4924 inhibited human multiple myeloma (MM) cell proliferation with the EC_50_ values ranging from 25 to 150 nM. Also, MLN4924 possessed comparable activity toward the proteasome inhibitors bortezomib-resistant ANBL-6 subline and its bortezomib-sensitive parental cells. Moreover, MLN4924 displayed submicromolar activity (EC_50_ < 0.5 μM) toward CD138^+^ multiple myeloma cells, and revealed synergistic effect with doxorubicin, bortezomib and dexamethasone on MM.1S cells. More importantly, MLN4924 was well tolerated at doses up to 60 mg/kg 2× daily and could effectively decrease tumor growth in both an orthotopic and a subcutaneous mouse model of multiple myeloma 126. Continually, Gu and co-workers indicated that the efficacy of MLN4924 on MM cell lines was related to the inhibition of AKT and mTOR pathways *via* increasing the expression levels of REDD1. The combination of MLN4924 with bortezomib synergistically caused MM cell apoptosis, which overcame the prosurvival effects of growth factors 127.

### The roles of neddylation in lymphoma

Smith's group found that MLN4924 treatment in activated B cell-like (ABC) diffuse large B-cell lymphoma (DLBCL) could lead to accumulation of pIκBα, reduction in nuclear p65 content, decrease of NF-κB transcriptional activity and G1 phase arrest, finally causing cell apoptosis. Additionally, in germinalcenter B cell-like (GCB) DLBCL cells, treated with MLN4924 resulted in the increased cellular Cdt-1 and S phase arrest, consistent with cells ongoing DNA re-replication. Besides, MLN4924 treatment in mice with human xenograft tumors of ABC- and GCB-DLBCL suppressed NAE pathway biomarkers and tumor growth 128. Jia and co-workers reported that MLN4924 blocked protein neddylation, inactivated CRL in lymphoma cells 129. In addition, MLN4924 inhibited the proliferation of lymphoma cells through inducing G2 phase arrest, along with senescence or apoptosis. Biologically, MLN4924-induced senescence was connected with the expression of tumor suppressor p21/p27, while apoptosis induction on neddylation inhibition was regulated by intrinsic apoptotic signaling pathway.

### The roles of neddylation in ovarian cancer

Pan and co-authors reported that CRL4 components were upregulated in human ovarian cancer tissues, and the activity of CRL4^CDT2^ was related to ovarian cancer cell (OCC) proliferation and survival. MLN4924 treatment could result in DNA damage, cell cycle arrest and apoptosis, and make OCCs more sensitive toward other chemotherapeutic drugs. Silencing of CRL4 components Cul4A, Roc1/2 and DDB1 displayed dramatically inhibitory potency on OCCs similar to MLN4924 treatment, indicating that CRL4 inhibition was beneficial for the efficacy of MLN4924. Besides, CDT2 downregulation mimicked the biological efficacy of MLN4924 and led to accumulation of its substrate CDT1. MLN4924-induced DNA damage and apoptosis in OCCs were partly relieved by CDT1 depletion, implying that CRL4^CDT2^ inhibition and CDT1 accumulation were related to the genotoxic properties of MLN4924 130. Hong et al. found that neddylation inhibition with MLN4924 interrupted PTX-induced microtubule polymerization, and effectively neutralized PTX-mediated antimigration, antiproliferative and apoptotic effects in OCCs. Additionally, disrupting neddylation through silencing of UBC12/UBE2F reduced the anticancer activity of PTX in OCCs. However, in comparison to knockdown of UBC12 or UBE2F alone, downregulation of both NEDD8 E2s simultaneously did not reveal synergistic efficacy on PTX resistance in OCCs. These findings revealed that neddylation potentially regulated the mechanisms of PTX resistance in ovarian cancer 131.

### The roles of neddylation in bladder cancer

Bladder cancer (BC) is a common urinary system malignancy, and over 90% of cases of bladder cancer are bladder urothelial carcinoma (UC) 132. Huang's group explored the effects of MLN4924 on human UC cell lines (NTUB1, T24 and RT4). They found that MLN4924 exerted potent antiproliferative activity, suppressed migration and invasion as well as induced G2M phase arrest and apoptosis, along with activation of Bad, caspase-3/7, PARP and phospho-histone H2A.X, reduction of phospho-Bcl2 level. Importantly, MLN4924 exhibited evidently anticancer activity on xenografted UC tumors in SCID mice without obvious toxicity 133. Continually, Tian et al. reported that NEDD8 was upregulated in BC tissues and associated with poor clinical outcomes of BC patients *via* analyzing the Cancer Genome Atlas (TCGA) database. Silencing of NEDD8 caused apoptosis and G2 phase arrest. In addition, NEDD8 inhibition with MLN4924 effectively suppressed proliferation, invasion and migration of BC cells *in vitro* and inhibited growth and metastasis of tumors *in vivo* 134.

### The roles of neddylation in chondrosarcoma

Chondrosarcoma, as one of the most common primary bone malignancy, usually generates cartilage matrix, which has no response to conventional therapies 135. MLN4924 revealed significant cytotoxicity against chondrosarcoma cells and caused cell apoptosis by activation of caspase-3/7. In addition, MLN4924 could inhibit cell proliferation through decreasing the phosphorylation of histone H3 to induce G2/M phase arrest. Besides, MLN4924 dramatically suppressed the growth of chondrosarcoma tumors in a xenograft mouse model, accompanied by the stimulation of ER stress-related apoptosis, indicating that neddylation inhibition with MLN4924 was an attractively therapeutic approach for chondrosarcoma 136.

### The roles of neddylation in cervical carcinoma

Cervical cancer (CC) is the second most common cancer in women over the world. Huang's group found that MLN4924 suppressed human CC cell proliferation and induced apoptosis, followed by activation of apoptosis-related molecules, interference with cell cycle regulators and Bid phosphorylation interruption. Additionally, MLN4924 resulted in an endoplasmic reticulum stress response (CHOP, ATF-4 and caspase-4 activations) and expression of other cellular stress molecules (c-Jun and JNK activations). Moreover, MLN4924 not only inhibited the growth of CC xenografts in nude mice, but also improved cisplatin-induced growth inhibition of CC xenografts. Together, these results offered a rationale for clinical trials of MLN4924 in CC 137.

### The roles of neddylation in pancreatic cancer

Jia's group uncovered that neddylation inhibition with MLN4924 suppressed angiogenesis, leading to the inhibition of tumor growth and metastasis in pancreatic cancer 39. Mechanically, MLN4924 inhibited CRLs through cullin deneddylation, accompanied by the accumulation of tumor-related substrates of CRLs, which further caused the accumulation of RhoA, DNA damage response, cell cycle arrest and apoptosis. Additionally, the inactivation of CRLs through silencing of RBX1, was similar to antiangiogenic effect of MLN4924.

Based on the relationships between neddylation proteins and various cancers, we can observe that neddylation proteins are overactivated in various cancer types (Table 1), and neddylation inhibition through silencing of single target shows potential antitumor potency. In particular, the effects of neddylation proteins are mainly explored for lung cancer, liver cancer, glioblastoma, HNSCC, colorectal cancer, prostate cancer and ovarian cancer. In addition, although the potential effects of some neddylation proteins like NAE, NEDD8, RBX1 for the occurrence and development of many kinds of cancers have been clarified, the regulatory potency of other neddylation proteins like UBC12, UBE2F, DCN1 etc. in some cancers remains to be further explored. Moreover, even though targeting neddylation is a promising approach for cancers, it is still unknown which protein can be targeted for specific cancer treatment, accompanied by potent activity and low toxicity. Therefore, numerous efforts need to be conducted for investigating the therapeutical effects of neddylation proteins in different cancers, which may be beneficial for precision drug development.

## Role of neddylation on fibrotic diseases

Fibrotic diseases, such as advanced liver disease, pulmonary fibrosis, heart failure and kidney disease, are a major public health concern in the world. To date, none of examined treatment has been absolutely proved to delay or prevent the progress of fibrotic diseases. Thus, it is imperative to understand the potential pathogenesis of fibrosis in hope of discovering an effective therapeutic approach 138-140. Recent years, targeting neddylation for the treatment of fibrotic diseases have been obtained more and more attention. In 2017, Imanol et al. firstly reported that neddylation was overactivated in clinical and two mouse models of liver fibrosis, including CCl4 chronic administration and bile duct ligation 24. Treated with neddylation inhibitor MLN4924 effectively relieved liver injury, apoptosis, inflammation and fibrosis by targeting different hepatic cell types. Our latest report indicated that HZX-960, a DCN1-UBC12 interaction inhibitor, effectively relieved TGFβ-induced liver fibrotic responses, demonstrated by the decrease of collagen I and α-SMA expressions, and the increase of NRF2, HO-1 and NQO1 levels in two hepatic stellate cell lines (LX2 and HSC-T6). Similar effects were observed in CCl4-treated mice after HZX-960 treatment as well 141. Continually, Wang and co-workers discovered that DCN1-UBC12 interaction inhibitor DI-1859 could protect mice from acetaminophen-induced liver injury, related to the inactivation of CRL3 and the accumulation of its substrate protein NRF2 142. Ge's group found that MLN4924 could significantly inhibit the occurrence and development of bleomycin-induced pulmonary fibrosis at the primary inflammatory stage. Mechanically, MLN4924 suppressed neddylation of CRL, resulting in abolishing NF-κB responses, inhibiting MAPK activity and decreasing the secretion of MCP1-induced chemokines and TNF-α-elicited pro-inflammatory cytokines 23. Neddylation, as an emerging regulatory mechanism of cardiac development and pathological remodeling, is important for heart function and ventricular compaction 143, 144. Our group observed that DCN1 protein was obviously upregulated in isolated cardiac fibroblasts treated by Angiotensin (Ang) II and in mouse hearts after pressure overload. However, inhibition of DCN1 effectively reversed Ang II-induced cardiac fibroblast activation, which was associated with the inhibition of cullin 3 neddylation and the accumulation of its substrate protein NRF2 145. Besides, we also found that treated with DCN1 inhibitor could potentially relieved liver fibrosis in CCl4-induced mouse model 141. In contrast to the above findings, Lin and co-authors reported that the expression levels of UBE2M and UBE2F were evidently decreased in pancreatic tissues from chronic pancreatitis mice in comparison to control mice 146. Besides, chronic pancreatitis mice administrated with MLN4924 displayed higher fibrosis-related gene expression, such as α-SMA and TGFβ, compared with the control group. These results reveal that targeting neddylation is a novel and promising therapeutic strategy for fibrotic diseases, which may be organ dependent.

## Inhibitors of neddylation enzymes

Although neddylation enzymes are considered as potential targets for cancer treatment, most of them have not been targeted for drug design. Here, we comprehensively summarize the neddylation enzyme inhibitors, including NAE inhibitors, DCN1-UBC12 interaction inhibitors and Cul1/5 inhibitor. Specifically, we cover their clinical applications, biological activities, design strategy, binding modes and structure-activity relationship studies.

### NAE inhibitors

**Figure 5:**
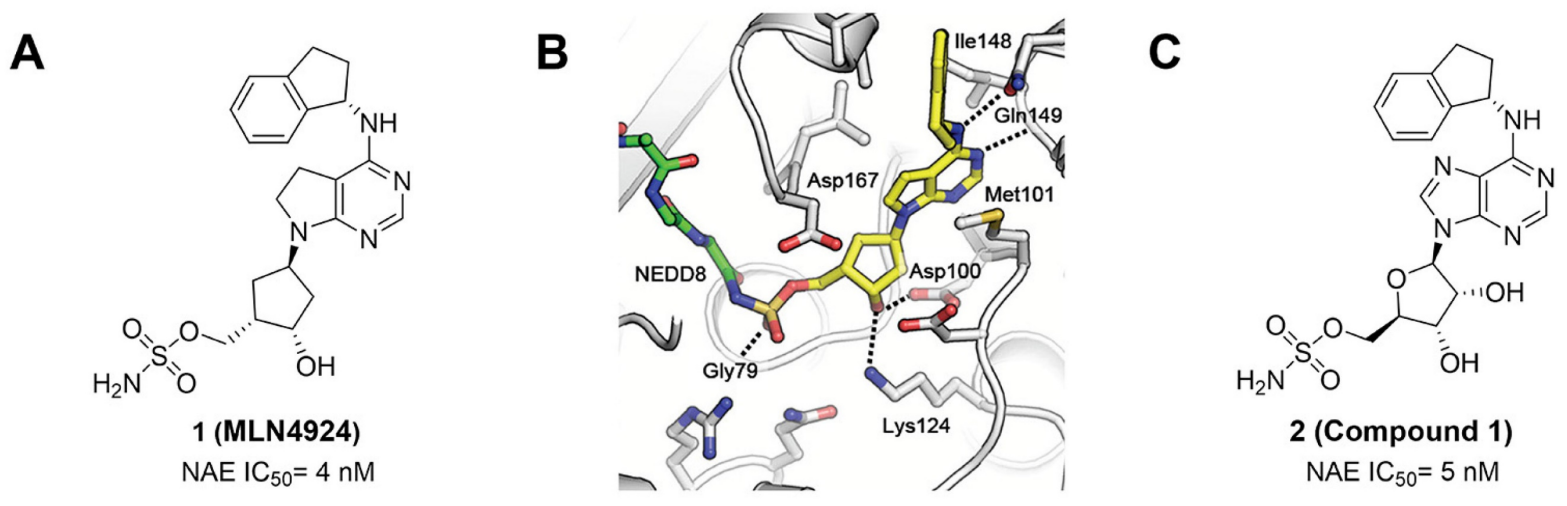
(A) The chemical structure of MLN4924 30. (B) The co-crystal structure of NAE (white), NEDD8 (green) and MLN4924 (yellow) (PDB: 3GZN) 147. Reproduced with permission 147. Copyright 2010, Elsevier Inc. (C) The chemical structure of molecule **2** 147.

MLN4924 (Pevonedistat), discovered in 2009 through high throughput screening, could selectively bind to the NAE active site to form a covalent NEDD8-MLN4924 adduct that prevented the formation of E2-NEDD8 thioester, therefore potently blocking whole neddylation modification (Figure 5A) 30. The co-crystal structure of NAE, NEDD8 and MLN4924 shows that there is a covalent NEDD8-MLN4924 adduct, which occupies the same sites as ATP and NEDD8 binding to the adenylation active site (Figure 5B). Especially, we observe several important interactions between MLN4924 and Gly79, Lys124, Asp100, Ile148 as well as Gln149 of NAE. These interactions are similar to those of NAE binding to the adenosine portion of ATP 147. Considering numerous preclinical data that neddylation inhibition with MLN4924 displayed robust anticancer efficacy with well-tolerated toxicity, several phase I/II/III clinical trials (https://clinicaltrials.gov/) were performed to evaluate the safety, pharmacokinetics (PK), pharmacodynamics and anticancer efficacy of MLN4924 alone or in combinations with chemo-/radiotherapy against patients suffering from AML, MS, melanoma, lymphoma and solid tumors (Table 2). Notably, two phase III trials of the combination of MLN4924 with azacytidine have been launched in patients with AML, MS and CMML. Additionally, MLN4924, as a chemo-/radiosensitizer, can sensitize several types of cancer cells toward chemotherapy agents and radiotherapy with different mechanisms, as shown in Table 3 and 4.

Smith and Petroski et al. disclosed that resistance to MLN4924 in HCT116 cells was correlated with hetereozygous mutations in the NEDD8-binding cleft of NAEβ and the adenosine triphosphate binding pocket 32, 148, 149. The specific mutations, including A171D, A171T, G201V, E204K, N209K and C324Y, caused decreased drug binding and slower rate of NEDD8-MLN4924 adduct formation. In contrast to MLN4924, compound **2** (Figure 5C), a N6-substituted adenosine sulfamate analog, was reported as a nonselective substrate-assisted inhibitor of canonical E1s and therefore was unsuitable for clinical application 147, 150. However, the NEDD8-compound **2** adduct bound more tightly to wild-type NAEβ and all mutant NAE enzymes than NEDD8-MLN4924 adduct in HCT116 cells, indicating that compound **2** might be a stronger inhibitor of mutant E1 enzyme in comparison to MLN4924. Continually, Xu et al. found two resistant R-K562_MLN_ and R-U937_MLN_ leukemia cells containing I310N and Y352H mutations in the NAE catalytic subunit UBA3 separately 151. These mutations led to the decreased efficacy of MLN4924, whereas offering necessary NAE function for leukemia cell growth. Surprisingly, the two MLN4924-resistant cells kept sensitive to compound **2**, probably due to its inhibitory effects on other E1 enzymes. Liu and co-workers reported that neddylation inhibition with MLN4924 was effective toward BCR-ABL mutational imatinib-resistant cells, and the self-renewal capacity of leukemia stem cells (LSCs) in CML. Biologically, MLN4924 caused CML cell G2/M phase arrest and apoptosis, regardless of their T315I mutation status in BCR-ABL. In addition, MLN4924 suppressed survival and self-renewal of human CML CD34^+^ cells, and inhibited LSCs in CML-bearing mice through accumulation of p27^kip1^ 85.

**Figure 6:**
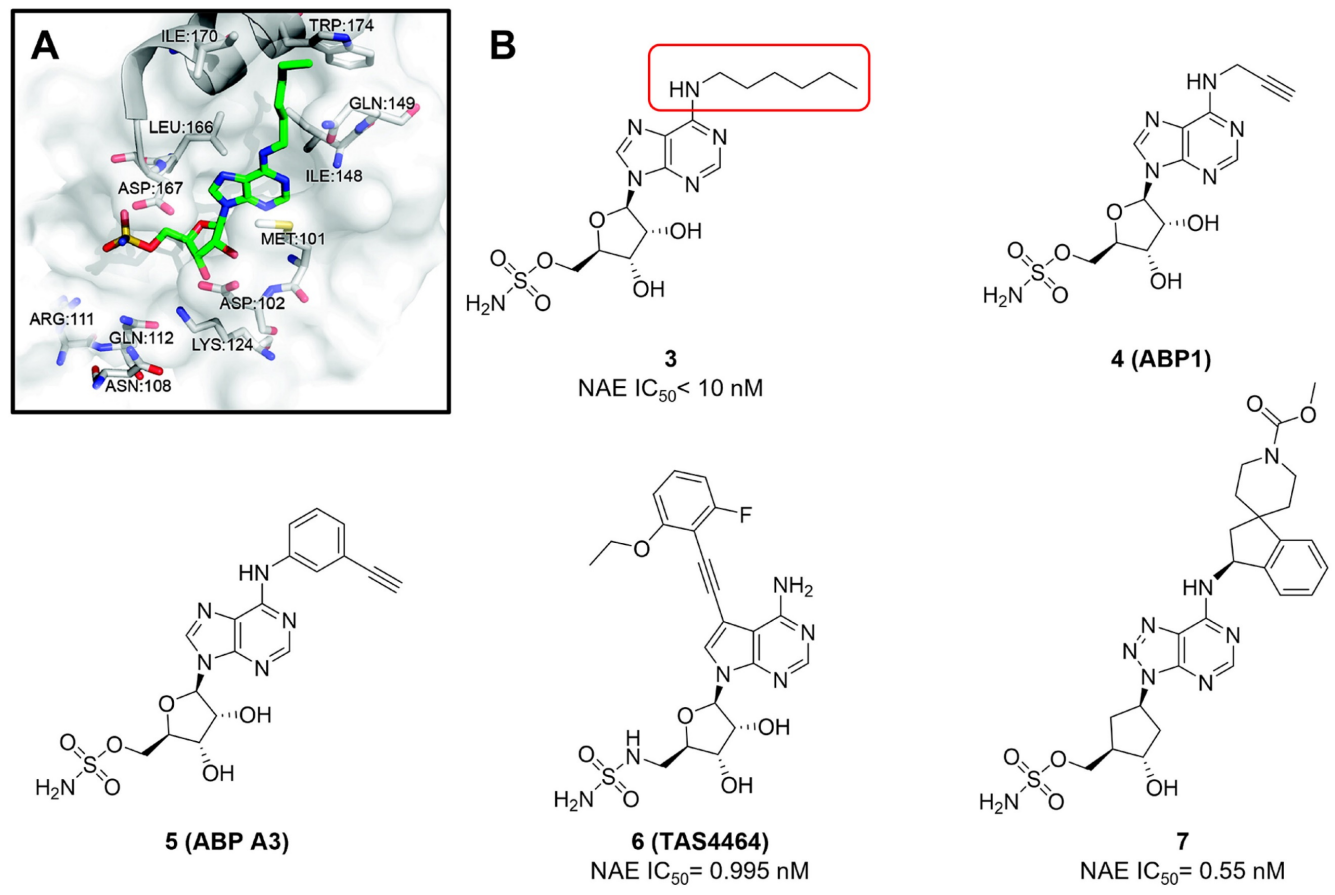
(A) The predicted binding modes of compound **3** (green) with NAE (white) 187. Reproduced with permission 187. Copyright 2011, American Chemical Society. (B) The chemical structures of MLN4924 analogues **3-7** 188-191.

Given that MLN4924 and compound **2** with similar structures possessed significant difference in their specificity for NAE, Lukkarila and co-workers next investigated the determinants of NAE selectivity based on compound **2** 187. Compounds with substituents of various size, flexibility and functionality in the purine C6 position were synthesized and evaluated against E1 enzymes NAE, ubiquitin activating enzyme (UAE) and SUMO-activating enzyme (SAE), resulting in compound **3** tethering an n-hexyl group, which showed the strongest binding affinity against NAE (IC_50_< 10 nM) in tested compounds, and was highly selective for NAE over UAE and SAE (Figure 6). The structure-activity relationships (SARs) indicated that introduction of N-alkyl groups into the purine C6 position was beneficial for NAE specificity, whereas bulky or secondary N-alkyl substituents were not tolerated. The predicted binding modes reveals that the alkyl group of compound **3** well occupies the hydrophobic region formed by Ile148, Gln149, Ile170 and Trp174 (Figure 6A). Besides, analogue **3** exhibited potent antiproliferative activity toward K562 leukemia cells with the IC_50_ value of 160 nM, slightly weaker than that of MLN4924 (IC_50_= 108 nM). Based on compound **2**, Statsyuk's group developed a pan-E1 inhibitor** 4** (Figure 6), which could decrease the expression levels of Ub, NEDD8, SUMO1/2/3 and Ufm1 conjugates in A549 cells. In contrast, compound **5** was reported as a dual inhibitor of ubiquitin- and NEDD8-activating E1 enzymes, and showed modest cytotoxicity against A549 cells with the IC_50_ value of 2.5 μM (Figure 6). Additionally, compound **5** could effectively induce A549 cell apoptosis, as demonstrated by the detection of cleaved PARP and Annexin V-positive cells 188, 189.

From library screening and structure-based design, compound TAS4464 (**6**) was identified and displayed more potent and more selective against NAE than MLN4924 (Figure 6) 190. Compound **6** could effectively inhibit cullin neddylation, further inducing the accumulation of CRL substrates. Additionally, treated with compound **6** showed strongly and broadly antiproliferative activity toward cancer cell lines as well as patient-derived tumor cells, and revealed a wider therapeutic index in comparison to MLN4924. Meanwhile, Ohkubo's group reported that compound **6** could cause growth arrest and cell death in tested 14 multiple myeloma cell lines 192. It also impaired the activities of NF‐κB transcription factors p65 and RelB and reduced the expression of NF‐κB target genes, indicating that derivative **6** inhibited both the canonical and non-canonical NF‐κB pathways. Moreover, compound **6** synergistically improved the anticancer efficacy of MM chemotherapies bortezomib, daratumumab, elotuzumab and dexamethasone. Considering these favorable preclinical findings, a phase 1 study was conducted to explore the efficacy of compound **6** in patients with advanced/metastatic solid tumors 193. The results indicated that abnormal liver function test (LFT) changes and gastrointestinal effects were the most common adverse events (AEs). Dose-limiting toxicities with 56-mg/m^2^ weekly dosing occurred in 1/5 patients; five patients possessed grade ≥ 2 abnormal LFT changes. These severe AEs terminated the applications of compound **6** in clinical trial.

Recently, a structural hopping strategy was performed for optimizing the deazapurine skeleton and the solvent interaction region of MLN4924, providing molecule **7** tethering a pyrimidotriazole scaffold (Figure 6) 191. Compound **7** was a potent NAE and UAE dual inhibitor with the IC_50_ values of 0.55, 66.84 nM separately. Additionally, derivative **7** possessed robust antiproliferative activity against ten tested cancer cell lines with submicromolar activity. Besides, analogue **7** displayed higher plasma concentrations and better PK properties in mice compared to its parental compound MLN4924. Mechanically, treated with compound **7** effectively suppressed the neddylation pathway and increased CRL substrates. Importantly, molecule **7** revealed potent antitumor potency and a good safety profile in MV-4-11 and HCT-116 xenograft models.

**Figure 7:**
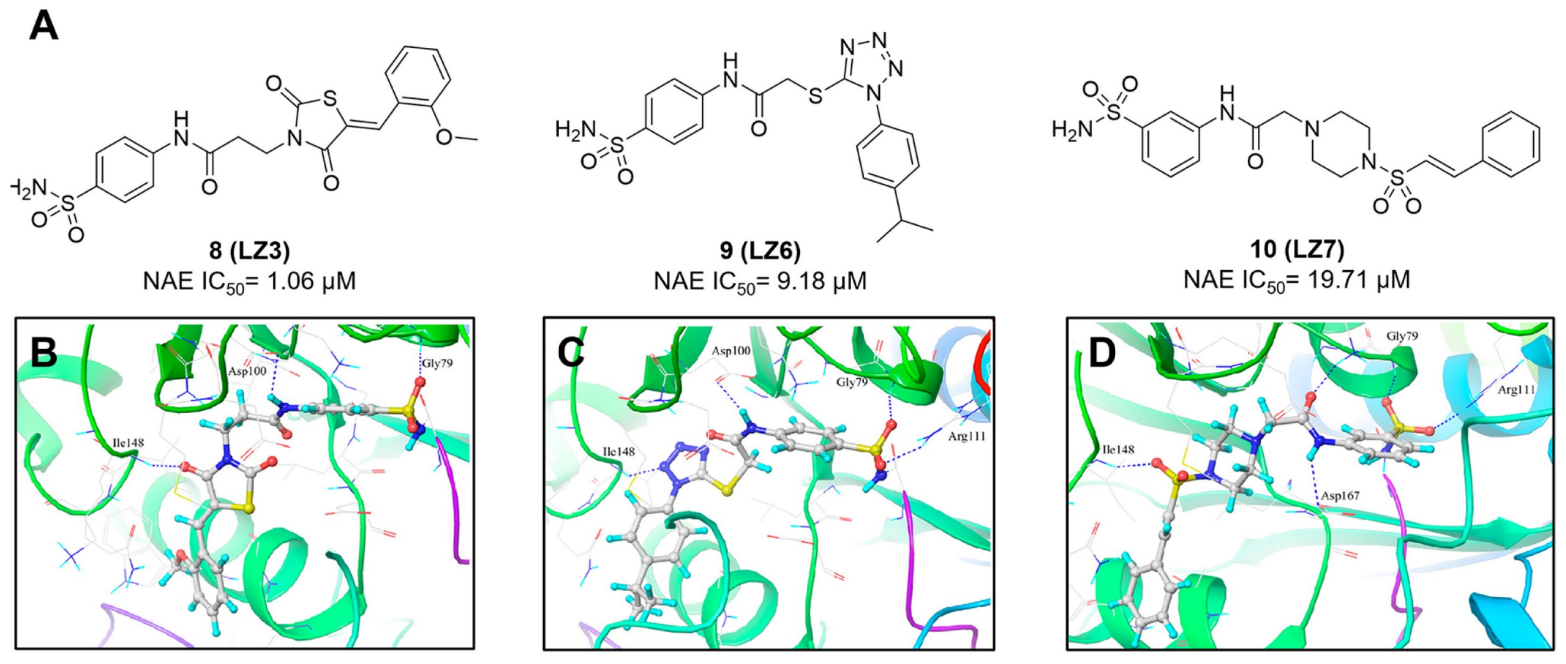
(A) The chemical structures of sulfamoyl-substituted analogues **8-10**. (B-D) The corresponding binding modes of compounds **8-10** with NAE, respectively 194. Reproduced with permission 194. Copyright 2014, American Chemical Society.

With the aim of identification of novel covalent NAE inhibitors, Zhang et al. conducted a combined virtual screen strategy through using both structure-based pharmacophore modelling and covalent docking, resulting in the discovery of sulfamoyl-substituted compounds **8-10** with the IC_50_ values of 1.06, 9.18, 19.71 μM separately (Figure 7A) 194. The docking studies exhibits that the sulfamoyl moiety of three molecules can form covalent bonds with Gly79 of NAE separately (Figure 7B-D), similar to the binding modes of MLN4924. In particular, compound **8** presented potent *in vitro* anticancer activity toward Bel-7402, Caco-2 and MCF-7 cells with the IC_50_ values ranging from 12.3 to 29.5 μM. Taken together, these novel NAE inhibitors can be regarded as hit compounds for further optimization.

**Figure 8:**
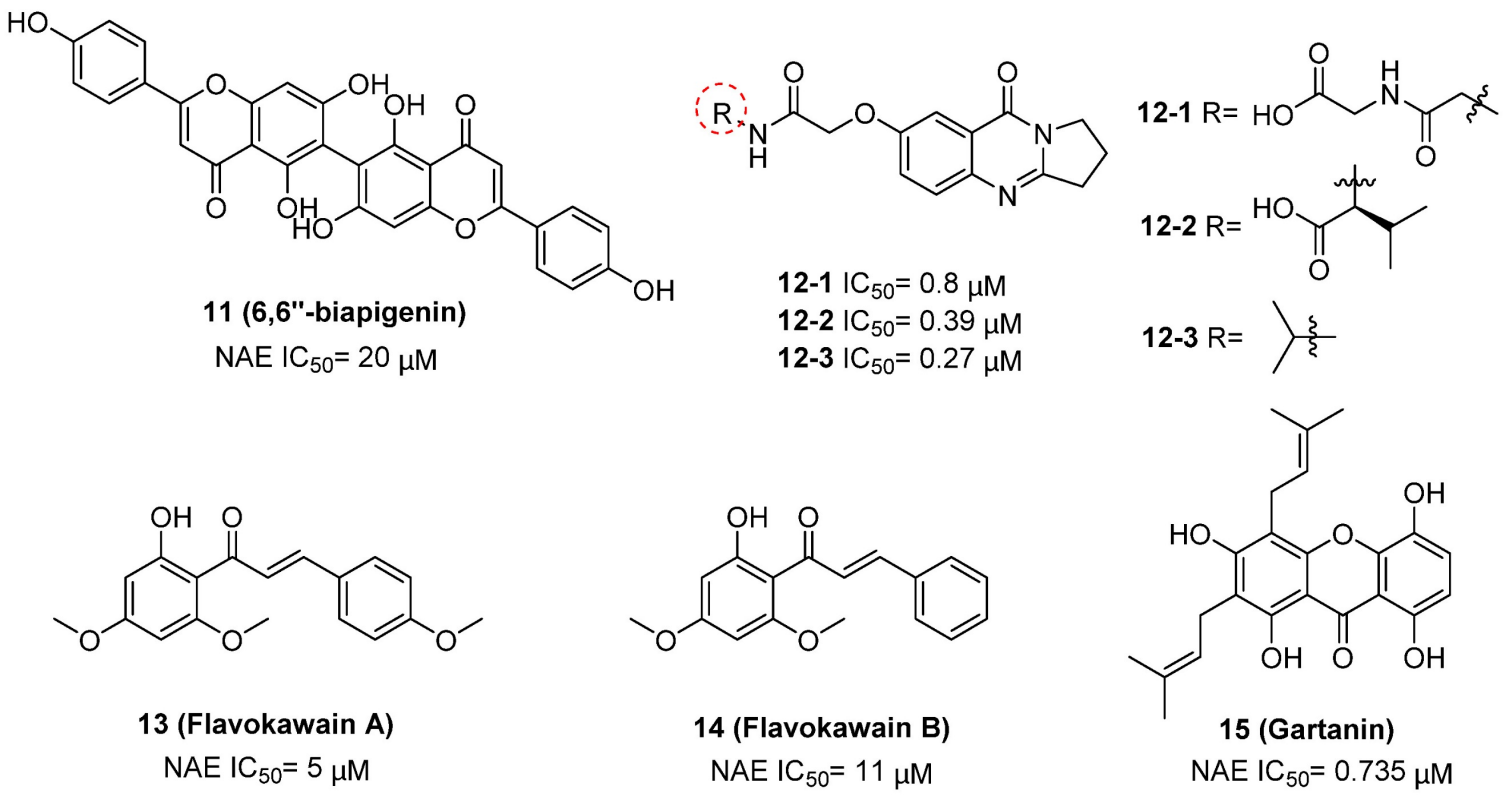
The chemical structures of natural products **11-15** 195-200.

Compound **11** was found as a new NAE inhibitor *via* virtual screening using a chemical library of natural product and natural product-like structures (Figure 8) 195. It revealed modest binding activity on NAE with the IC_50_ value of 20 μM. Treated with compound **11** could effectively inhibit NAE activity in colorectal adenocarcinoma Caco-2 cells with the concentration of 25 μM. In contrast to MLN4924, molecule **11** cannot form a covalent adduct with NEDD8. Similarly, Leung et al. discovered the dipeptide-conjugated deoxyvasicinone analogue **12-1** as a noncovalent NAE inhibitor through virtual screening of about 90,000 molecules from the ZINC database of natural products (Figure 8) 196. Densitometry analysis indicated that analogue **12-1** significantly suppressed NAE activity with the IC_50_ value of 0.8 μM. Also, compound **12-1** was highly selective for NAE over analogous E1 enzymes UAE and SAE at the concentration of 12.5 μM. Besides, derivative **12-1** showed moderate antiproliferative activity against Caco-2 cells (IC_50_= 10 μM, 48 h), which might be partly associated with the suppression of NAE activity, further causing the accumulation of CRL substrates like p27^kip1^. Encouraged by the efficacy of compound **12-1**, Leung's group used the pharmacophore screening to further identify analogues **12-2** and **12-3** as more potent NAE inhibitors with the IC_50_ values of 0.39, 0.27 μM, respectively 197. **12-2** and **12-3** showed similarly biological profiles compared with molecule **12-1**. Compounds **13** and **14** (Figure 8), as naturally occurring chalcone discovered in the Kava plant, were new NAE inhibitors and suppressed NEDD8 to both Cul1 and UBE2M in prostate cancer cells and UBE2M neddylation 198, 199. Molecular docking study and the cellular thermal shift assay revealed that both compounds could directly bind to the NAE regulatory subunit APP-BP1, which was responsible for anti-prostate cancer activity. Mechanically, they interacted with APP-BP1, leading to the deneddylation of Cul1 and UBE2M, the decreased activity of SCF^SKP2^ complex and SKP2 ubiquitination and degradation, as well as the activation of caspase mediated apoptotic pathway. Gartanin (**15**), a 4-prenylated xanthone, was identified from the purple mangosteen fruit, and showed potential enzymatic activity on NAE with the predicted IC_50_ value of 0.735 μM (Figure 8) 200. In-depth mechanism research indicated that compound **15** could deprive NEDD8 from UBC12 and Cul1, which changed the specific SCF ubiquitin ligases and finally resulted in cell growth inhibition and autophagy.

**Figure 9:**
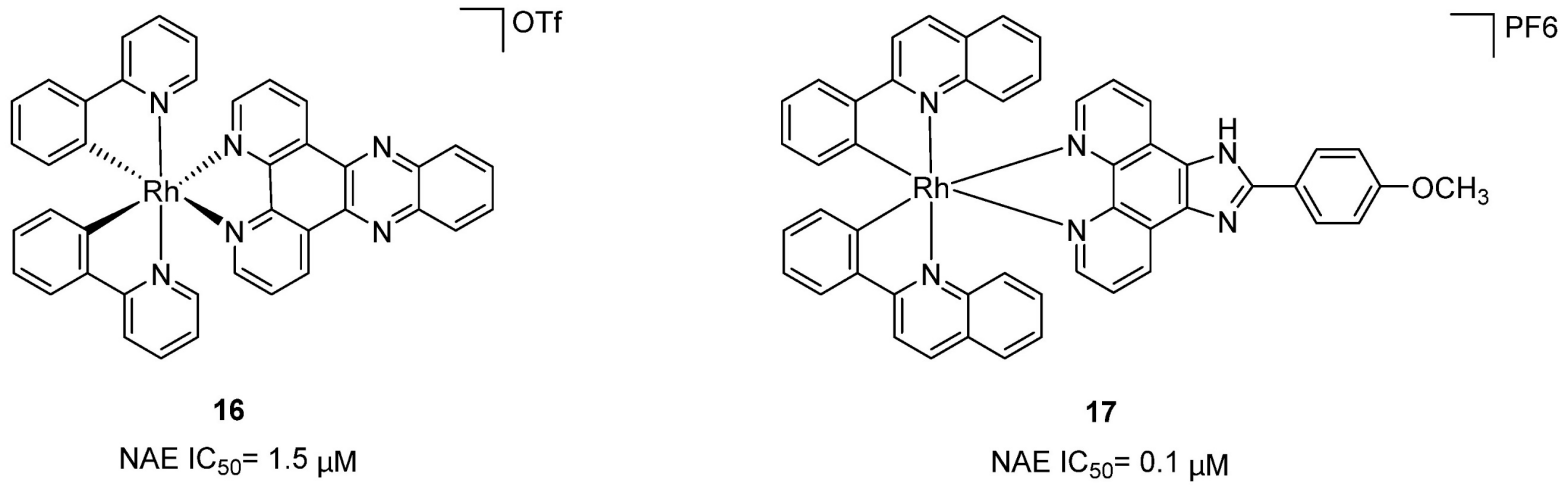
The chemical structures of metal-based NAE inhibitors **16-17** 201, 202.

Zhong et al. designed and synthesized a novel class of rhodium(III) complexes as NAE inhibitors, resulting in molecule **16** (R_1_, R_2_, R_3_= H) with the IC_50_ value of 1.5 μM (Figure 9) 201. The SARs showed that replacement of the hydrogen at R_1_, R_2_ and R_3_ with other substituents caused weaker inhibitory activity. Also, the metal center played a critical role for recognizing protein binding site. Derivative **16** exerted potent antitumor effects on Caco-2 cells with the IC_50_ value of 0.3 μM, ten folds stronger than that of MLN4924 in a parallel experiment, which might be related to the inhibition of CRL substrate degradation, increase of p27 protein level and reduction of NF-kB pathway. Continually, Zhong and co-authors reported the rhodium(III) complex [Rh(phq)_2_(MOPIP)]^+^ (**17**) as a potent and selective ATP-competitive NAE inhibitor with the IC_50_ value of 0.1 μM using an AlphaScreen assay, about 15 folds more active in comparison to compound **16** (Figure 9) 202. In addition, compound **17** could inhibit Caco-2 cell proliferation with the IC_50_ value of 4.3 μM. The preliminary SARs study indicated that the 1*H*-imidazo[4,5-f][1,10]phenanthroline moiety and methoxy group in compound **17** were beneficial for binding affinity. Besides, changing the Rh(III) center of **17** to Ir(III) dramatically decreased NAE inhibitory activity. Furthermore, complex **17** could block the degradation of CRL substrates NRF2 and CDT1, partly linked to NAE inhibition.

**Figure 10:**
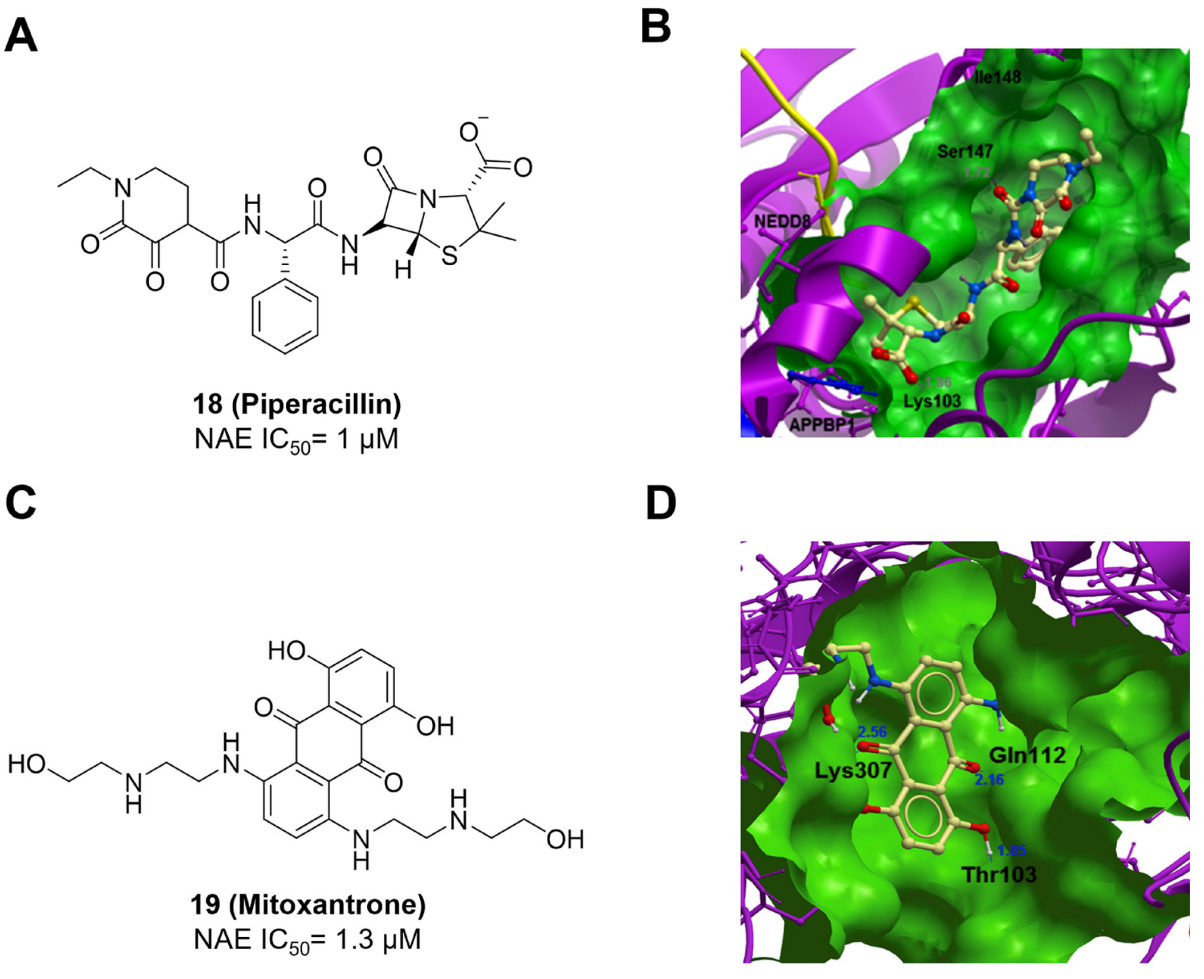
(A) and (C) The chemical structures of FDA-approved drugs **18-19**. (B) The binding modes of compound **18** (yellow) with NAE (green). Reproduced with permission 203. Copyright 2014, Elsevier Masson SAS. (D) The binding modes of compound **19** (yellow) with NAE (green) 203, 204. Reproduced with permission 204. Copyright 2018, Elsevier Masson SAS.

Through the structure-based virtual screening using an FDA-approved drug library of around 3000 molecules, Zhong et al. identified piperacillin (**18**) as an effective NAE inhibitor with the IC_50_ value of 1 μM (Figure 10A) 203. Compound **18** exhibited highly selective for NAE over SAE and ubiquitin E2 enzyme UBC10 in Caco-2 cells. Also, treated with **18** caused the accumulation of CRL substrate p27^kip1^. The docking study presents that the binding pose of molecule **18** in NAE overlaps markedly with that of ATP (Figure 10B), indicating that **18** may serve as a reversible ATP-competitive inhibitor of NAE. Mitoxantrone (**19**) was also reported as an ATP-competitive reversible inhibitor of NAE. It could inhibit NAE-mediated UBC12-NEDD8 conjugation in Caco-2 cells with the IC_50_ value of 1.3 μM (Figure 10C) 204. In addition, compound **19** displayed higher selectivity against NAE over SAE and UAE. The molecular docking study reveals that the hydroxyl group of **19** makes a hydrogen bond interaction with Thr103 (Figure 10D). One of the carbonyl groups acts as a hydrogen bond acceptor with Gln112, whereas the other carbonyl group interacts with the sidechain of Lys307. Besides, one of the alkyl chains of derivative **19** extends out the solvent region, while the other is located in the binding pocket. Biologically, analogue **19** induced Caco-2 cell apoptosis *via* regulation of cullin-dependent substrates.

**Figure 11:**
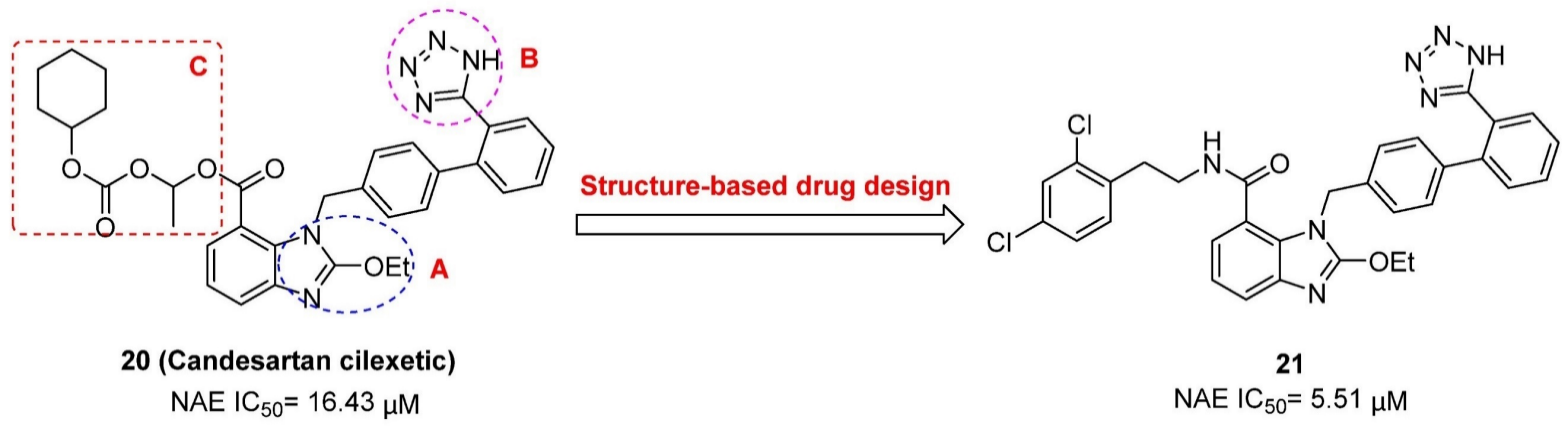
The chemical structures of FDA-approved drug **20** and its analogue** 21** 205, 206.

To discover new NAE inhibitors, Jia's group screened 1331 approved drugs by evaluating the Cul1-NEDD8 inhibition, yielding the hit compound candesartan cilexetil (**20**) with the IC_50_ value of 16.43 μM (Figure 11) 205. Compound **20** bearing a benzimidazole moiety could obviously inhibit A549 cell proliferation with the IC_50_ value of 63.93 μM in CCK-8 cell viability assay and induce A549 cell apoptosis. Besides, molecule **20** not only suppressed neddylation pathway through determining the formations of Cul1-NEDD8 and UBC12-NEDD8 adducts, but ATP-competitively inhibited neddylation modification. The antitumor activity *in vivo* presented that molecule **20**-treated with 60 or 30 mg/kg group effectively inhibited the growth of A549 xenograft tumors. To overcome the defect of structurally easy hydrolysis and enhance the antitumor efficacy of derivative **20**, further structural modifications were performed *via* focusing on the region A, B and C of compound **20**, providing 42 new derivatives with a benzimidazole skeleton 206. The SARs indicated that the benzimidazole and tetrazole groups were beneficial for neddylation inhibitory activities. The introduction of (cyclo) alkyl, benzoalicyclic and heterocyclic groups into region C caused decreased activities, whereas the replacement of phenyl groups improved biological activities. Among synthesized compounds, molecule **21** (Figure 11) possessed the strongest neddylation inhibitory activity with the IC_50_ value of 5.51 μM, about 3 folds more active than compound **20**. Additionally, analogue **21** showed potent antiproliferative activity toward A549 cells with the inhibition rate of 83.9% at 100 μM. Besides, molecule **21** could induce A549 cell apoptosis and senescence. Notably, compound **21** not only inhibited the growth of A549 xenograft tumors on administration of intraperitoneal injection (30 mg/kg), but also displayed potent anticancer potency *via* oral administration (60 mg/kg).

**Figure 12:**
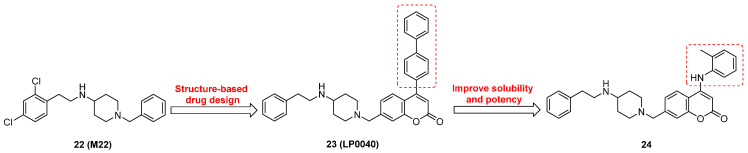
The chemical structures and design strategy of chromen-based NAE inhibitors **22**-**24** 207-209.

Lu and co-workers performed structure-based virtual screening of a compound library comprising 50,000 molecules, resulting in the identification of compound **22** (Figure 12) as a selective and reversible NAE inhibitor 207. Next, a dose-dependent decrease of the intensity of UBC12-NEDD8 bands was determined after compound **22** treatment. The results indicated that treated with molecule **22** with the concentration of 3.33 μM evidently suppressed the formation of UBC12-NEDD8. Additionally, derivative **22** exerted moderate *in vitro* anticancer effects on Caco-2, SK-OV-3, K562, AGS, and BXPC-3 cell lines with the GI_90_ values of 12.7, 13.2, 8.98, 14.9, and 26.6 μM separately. Besides, analogue **22** blocked neddylation and decreased the degradation of related substrates, leading to cell apoptosis. Furthermore, compound **22** effectively inhibited tumor growth in the nude mice xenograft model without obvious toxicity in the zebrafish model.

Based on compound **22**, a new class of 4-substituted 2H-chromen-2-one analogues were designed and synthesized through mainly focusing on the 4-position of 2H-chromen-2-one 208. In comparison to molecule **22**, most synthesized compounds displayed increased antiproliferative activity toward A549, K562, HCT116, SKOV-3, BxPC-3 and AGS cells with micromolar activity. The SARs studies disclosed that 4-hydrophobic substituents could benefit inhibitory activity, while 4-hydrophilic substituents decreased activity. Additionally, introduction of ethynyl groups into 4-position of 2H-chromen-2-one contributed to the enhancement of the antiproliferative activity. Especially, compound **23** (Figure 12) bearing a biphenyl group indicated the strongest biological activity against AGS cells (IC_50_= 0.76 μM) and low toxicity toward normal HEK293T, HUVEC and L-02 cells with the LC_50_ values of 12.93, 10.69, 13.54 μM separately. Derivative **23** was found as a NAE/UAE dual inhibitor and could inhibit the formation of UBC12-NEDD8 at the concentration of 0.37 μM. Besides, compound **23** effectively induced AGS cell G1 phase arrest and apoptosis.

Continually, to improve water solubility of compound **23**, Lu et al. introduced various aliphatic amino or aromatic amino into the 4-position of 2H-chromen-2-one, offering a new class of 2H-chromen-2-one analogues 209. In particular, compound **24** (Figure 12) exerted the strongest cytotoxicity on BxPC-3 cells with the IC_50_ value of 0.17 μM, more potent than compound **23** (IC_50_= 2.8 μM), and also indicated low toxicity toward normal cells. Compared to analogue **23**, compound **24** tethering a 4-aromatic amino group revealed better solubility at 340.73 μg/mL and its Log P value was 1.66, implying that it possessed a reasonable druglike property. Additionally, molecule **24** was a promising ATP-dependent NAE inhibitor and inhibited the formation of UBC12-NEDD8 at 0.56 μM. Moreover, derivative **24** effectively induced BxPC-3 cell apoptosis and played a synergistic effect with bortezomib on BxPC-3 cell growth inhibition.

**Figure 13:**
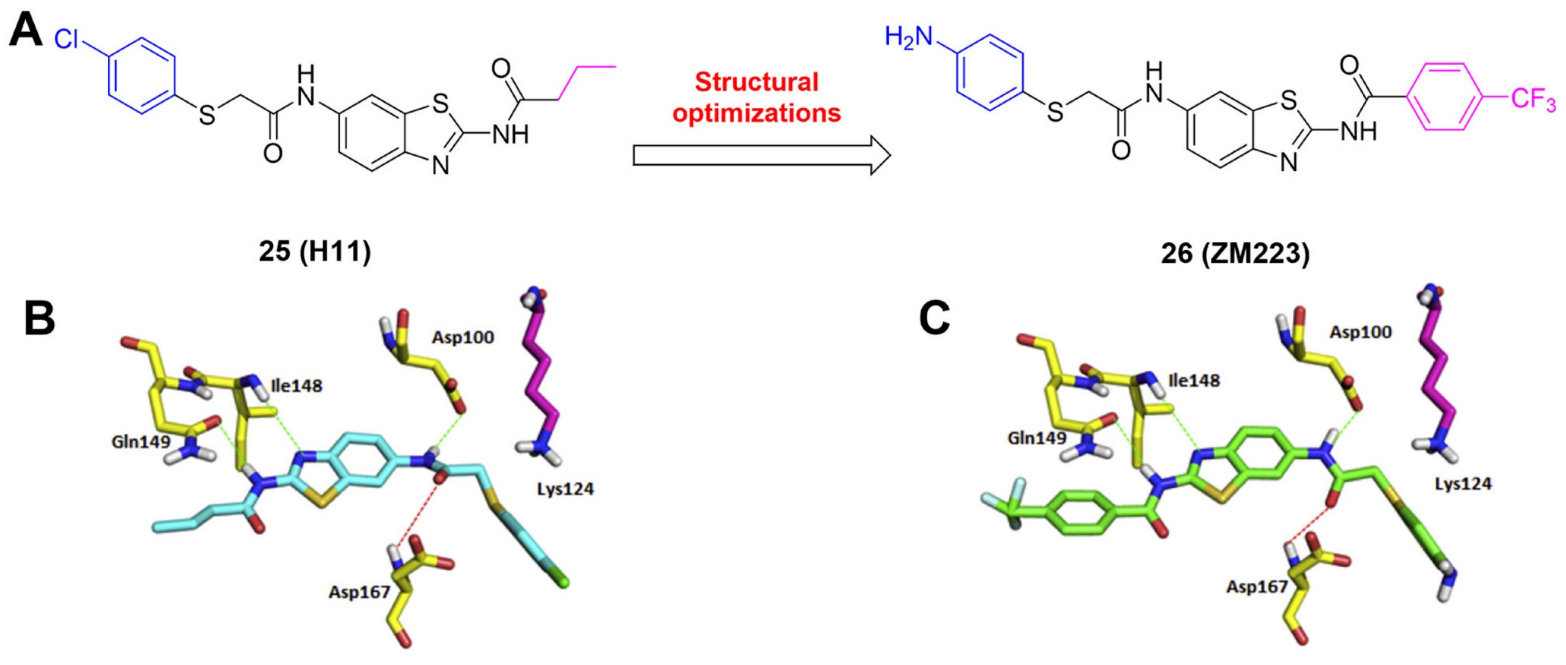
(A) The chemical structures of benzothiazole derivatives **25**-**26**. (B) Docking modes of analogues **25** (B) and **26** (C) with NAE 210. Reproduced with permission 210. Copyright 2017, Elsevier Masson SAS.

Miao's group conducted a target-based virtual screening to discover new NAE inhibitors, leading to compound **25** featuring a benzothiazole skeleton (Figure 13A) 210. Derivative **25** possessed strongly antiproliferative activity on HCT116 and U-2OS cells with the IC_50_ values of 2.12, 3.19 μM, respectively. Next, two round structural optimizations were performed *via* focusing on the non-hydrogen bonding sides of compound **25**, yielding compound **26**, which displayed significantly increased cytotoxicity toward HCT116 and U-2OS cells with the IC_50_ values of 0.1, 1.22 μM separately. The docking study indicates that both compounds make hydrogen bond interactions with Asp100, Ile148, Gln149 and Asp167 (Figure 13B-C). Biologically, derivative **26** could effectively suppress NEDD8 protein and subsequently elevate the UBC12 level.

**Figure 14:**
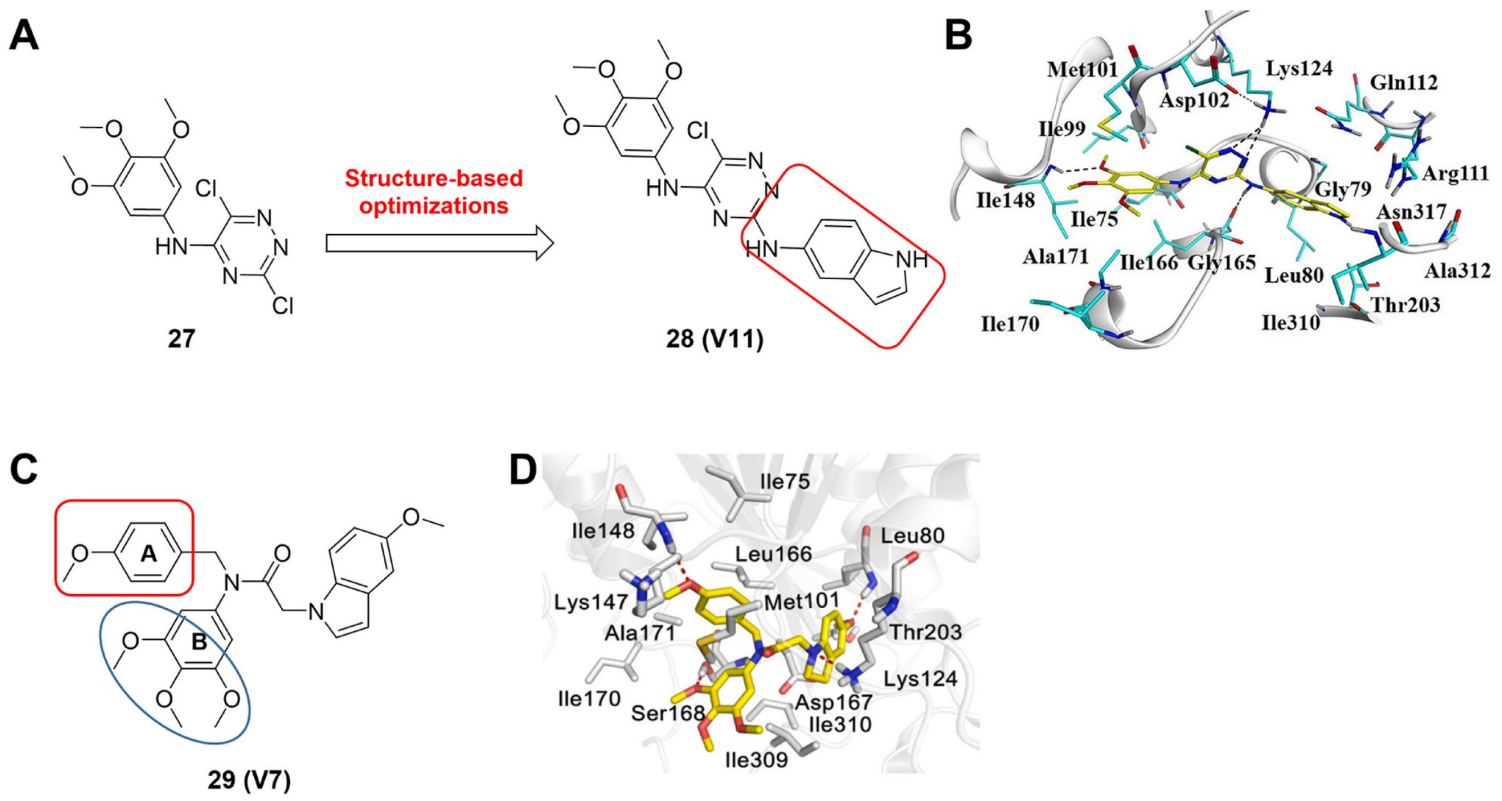
(A) The chemical structures and design strategy of indole-based NAE inhibitors **27**-**28**. (B) Docking modes of compound **28** with NAE. Reproduced with permission 211. Copyright 2020, Elsevier Ltd. (C) The chemical structure of indole-based NAE inhibitor **29**. (D) Docking modes of compound **29** with NAE 211, 212. Reproduced with permission 212. Copyright 2020, Elsevier Inc.

Zhang's group screened their in-house structurally diverse molecular library, providing triazine-based compound **27** as a hit for NAE inhibition (Figure 14A) 211. Further modifications were focused on the 3-position of triazine moiety in **27** *via* introducing various aromatic groups and indole groups. Afterwards, molecule **28** featuring an indole group was obtained and displayed modest anticancer activity toward MGC803, PC3, MCF-7 cells with the IC_50_ values of 8.22, 9.28, 9.64 μM separately. Compound **28** evidently inhibited the activity of NAE, demonstrated by the decrease of UBC12-NEDD8 conjugation in MGC803 cells with the EC_50_ value of 3.56 μM. The molecular docking indicates that derivative **28** forms hydrogen bonds with Lys124, Ile148 and Gly165, respectively (Figure 14B). Additionally, the indole ring makes strongly electrostatic interactions with the surrounding residues Gly79, Arg111, Gln112 and Asn317, and hydrophobic interactions with Leu80, Thr203, Ile310 and Ala312. Besides, analogue **28** caused MGC803 cell G2/M phase arrest and induced apoptosis. Continually, Zhang's group synthesized a new array of indole-based compounds mainly through the modifications of rings A and B (Figure 14C) 212. Particularly, compound **29** exhibited the strongest biological activity toward MGC803, PC3, EC109 cells with the IC_50_ values of 1.59, 3.56, 14.52 μM separately. Also, molecule **29** interacted with NAE regulatory subunit to suppress UBC12 neddylation in MGC803 cells. The docking study discloses that compound **29** well occupies the active cavity of NAE (Figure 14D). Hydrogen bond interactions are formed between 4-methoxyl-phenyl group and Ile148, 3,4,5-trimethoxyl-phenyl group and Serl68. The 5-methoxy-indole group also makes hydrogen bond interactions with Leu80 and Lys124. Mechanism research indicated that analogue **29** not only resulted in MGC803 cell G2/M phase arrest, but also inhibited neddylation and MAPK pathways, further leading to activated extrinsic and intrinsic apoptosis pathways.

**Figure 15:**
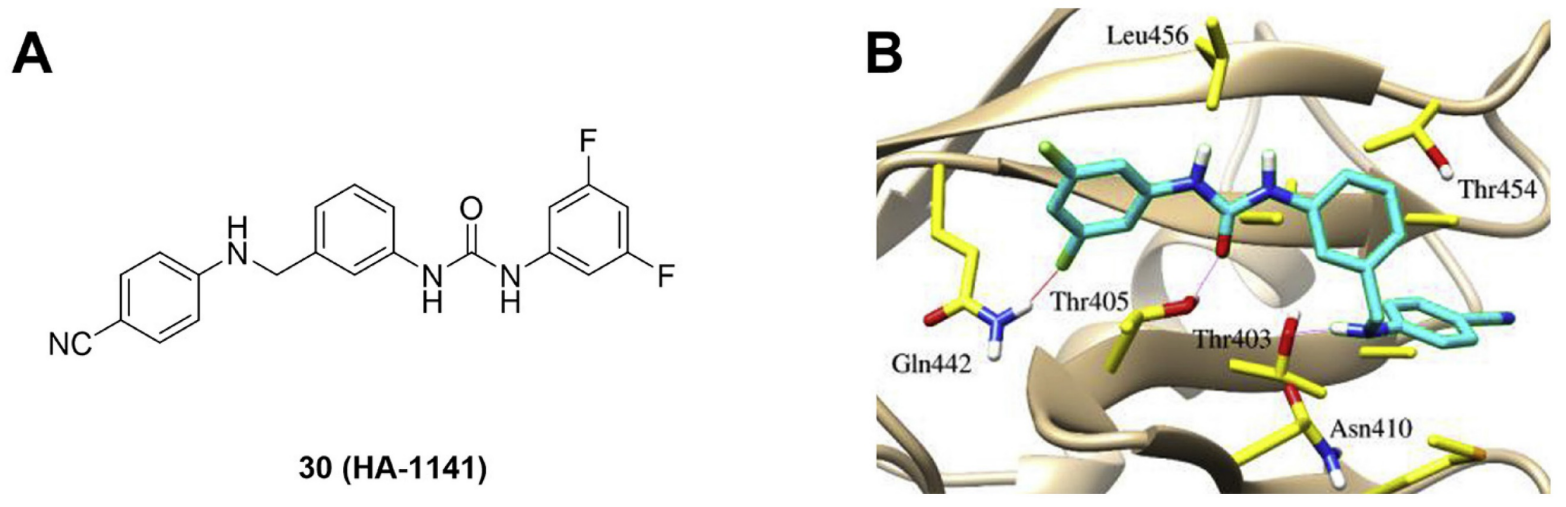
(A) The chemical structure of urea derivative-based NAE inhibitor **30**. (B) Docking modes of compound **30** with NAE 213. Reproduced with permission 213. Copyright 2021, Elsevier B.V.

In 2021, Sun's group identified the NAE inhibitor **30** (HA-1141) through virtual screening and further modifications, which significantly suppressed neddylation of cullins 1-5 (Figure 15A) 213. The docking study shows that compound **30** well occupies the active pocket consisting of amino acids Gln 442, Thr405, Thr403, Asn410, Leu 456 and Thr454 (Figure 15B). Especially, derivative **30** forms two critical hydrogen bonds with Thr403 and Thr405, respectively. Additionally, a halogen bond is found between Gln442 and a fluorine atom in compound **30**. In contrast to MLN4924, compound **30** could effectively inhibit the growth of lung cancer cells *in vitro* and tumor growth *in vivo* xenograft lung cancer models *via* suppressing neddylation and triggering ER stress.

**Figure 16:**
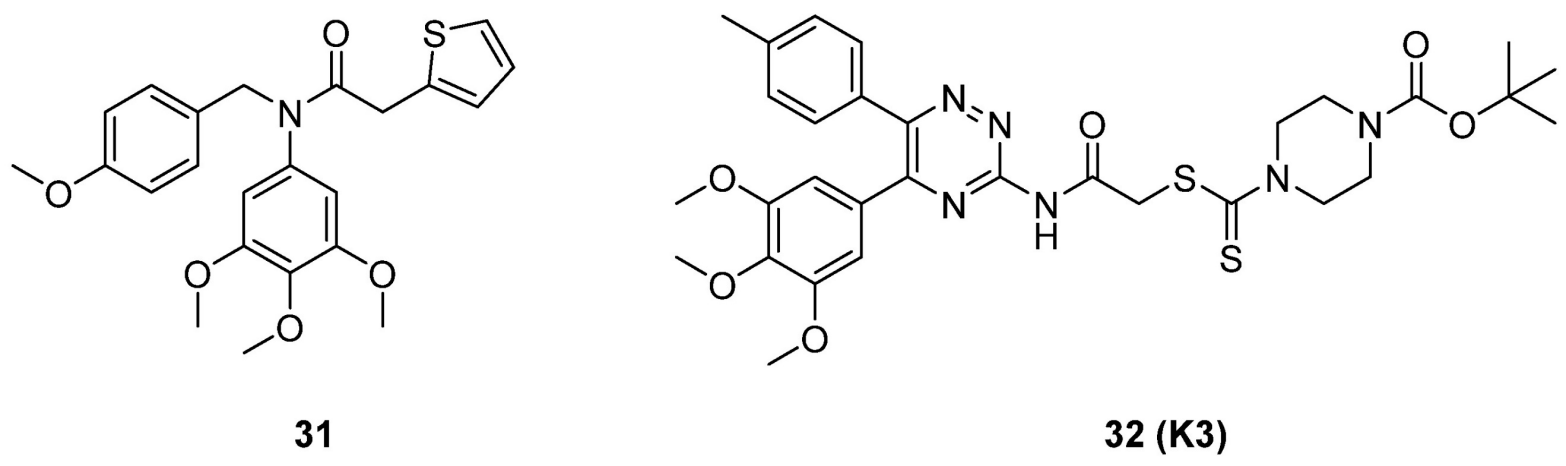
The chemical structures of NAE activators **31-32** 214, 215.

Considering that neddylation affecting many pathological processes, NAE activators also have been reported recent years. In 2020, Fu et al. found a new series of amide derivatives, indicating potent antiproliferative activity toward MGC803, MCF-7 and PC-3 cell lines 214. In particular, molecule **31** displayed the strongest effects on inhibiting MGC803 growth with the IC_50_ value of 94 nM (Figure 16). Further evaluation revealed that compound **31** not only caused MGC803 cell arrest at G2/M phase, but induced MGC803 cell apoptosis through intrinsic and extrinsic pathways. Further mechanism study showed that compound **31** could activate NAE1-UBC12-Cullin1 neddylation through binding NAE protein straightly, leading to the degradation of anti-apoptosis protein IAPs. Besides, compound **31** could significantly suppress tumor growth in xenograft models without noticeable toxicity *in vivo*. Compound **31** was the first molecule reported for activating NAE. Continually, their group developed a new class of 1,2,4-triazine-dithiocarbamate derivatives as anti-cancer agents in 2021, of which the representative compound **32** (Figure 16) possessed the most potent cytotoxicity on MGC-803, PC-3 and EC-109 cell lines with the IC_50_ values of 2.35, 5.71 and 10.1 μM separately, more active compared to the positive control 5-Fu 215. In addition, compound **32** could enhance the neddylation of MGC-803 and HGC-27 cell lines through activating NAE, causing the degradation of anti-apoptosis proteins c-IAP and YAP/TAZ, which further induced cell growth inhibition and apoptosis. These findings indicates that the development of NAE activators may be also a new strategy for cancer treatment.

Overall, NAE modulators of different structural types have been reported, including N6-substituted adenosine sulfamate derivatives, sulfamoyl-substituted derivatives, flavonoid derivatives, chalcone derivatives, 4-prenylated xanthone derivatives, rhodium (III) complexes, beta-lactam derivatives, anthracenedione derivatives, benzimidazole derivatives, 2H-chromen-2-one derivatives, benzothiazole derivatives, indole derivatives and urea derivatives (Table 5). These modulators were developed mainly through high throughput virtual screening, structure-based drug design, structural hopping strategy, pharmacophore screening, structure-based virtual screening and drug repurposing. Generally, these reported NAE inhibitors revealed potent anticancer activity. Compared with other NAE inhibitors, the most N6-substituted adenosine sulfamate derivatives could covalently target NAE active site, and indicated stronger NAE inhibitory activity with low nanomolar level (Figure 5-6). However, due to the hetereozygous mutations (A171D, A171T, G201V, E204K, N209K and C324Y) in the NEDD8-binding cleft of NAEβ and the adenosine triphosphate binding pocket, these adenosine sulfamate-based NAE inhibitors partly occurred to resistance in some cancer cells. The development of new NAE inhibitors targeting those mutations may be a method for overcoming resistance. It is worth noting that Rh(III) complex **16-17** also showed obvious NAE binding activity. The specific structural features of the octahedral coordination geometry of the Rh(III) complex **16-17** made them form beneficial interactions with NAE, probably further resulting in enhanced activity and selectivity toward NAE over other closed-related E1 enzyme 201, 202. In addition to NAE inhibitors, Zhang's findings concerning NAE activators delivered us a new prospect for activating neddylation pathway in cancer treatment 214, 215. Besides, although numerous clinical trials are undergoing for exploring the anticancer effects of NAE inhibitor MLN4924, it is unclear whether NAE is an effective target for diseases clinically. Thus, more potent and selective NAE inhibitors with low toxicity and novel chemotypes remain to be urgently needed for investigating the clinical efficacy of targeting NAE.

### Inhibitors targeting DCN1-UBC12 interaction

**Figure 17:**
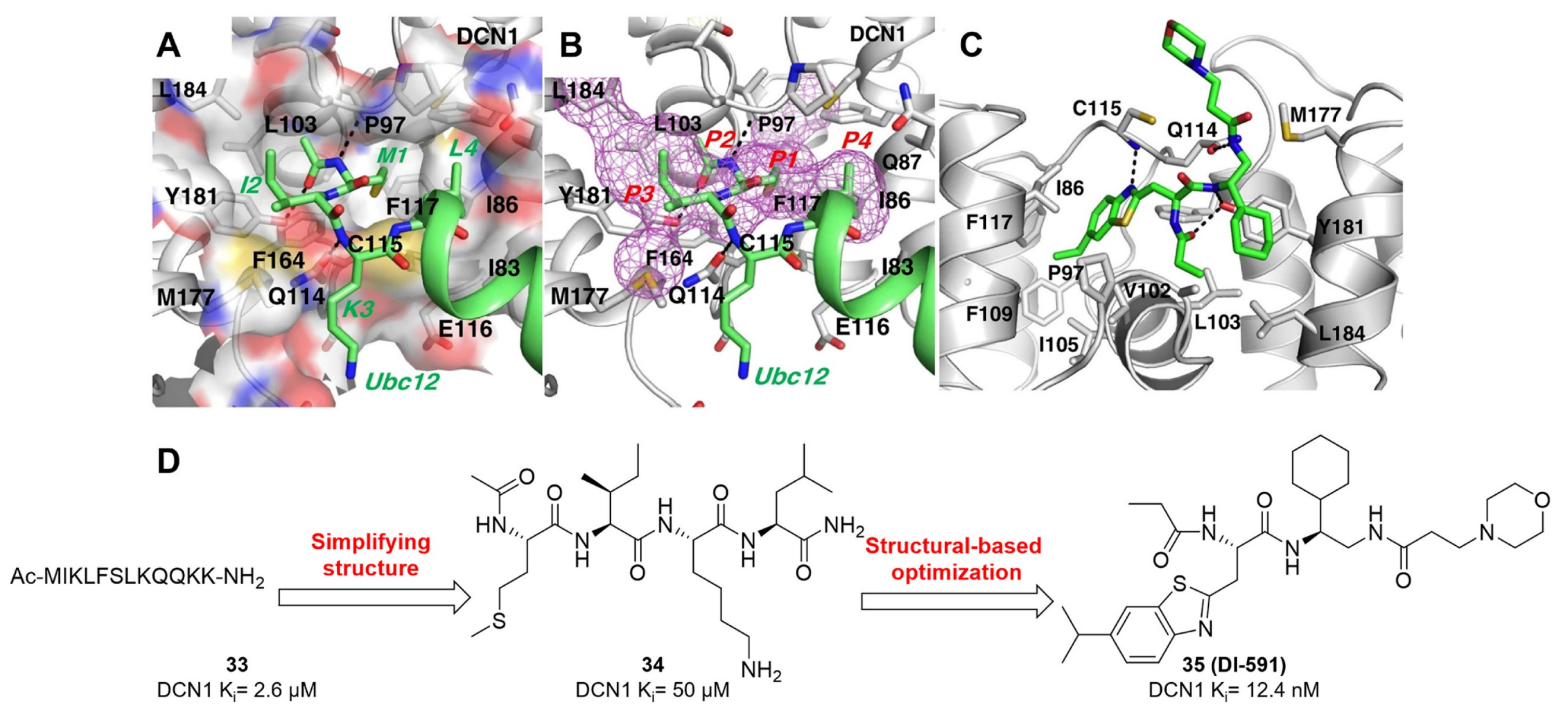
(A) The crystal structure of DCN1 complexed with UBC12 peptide (PDB: 3TDU). (B) Hydrophobic hotspots (purple mesh) at the UBC12 peptide binding site. (C) The co-crystal structure of DCN1 complexed with DI-591 (PDB: 5UFI). (D) The chemical structures and design strategy of peptidomimetic compounds **33**-**35** 218. Reproduced with permission 218. Copyright 2017, Nature Publishing Group.

Among the five DCN isoforms, human DCN1 is highly amplified in several tumors and essential in tumor formation, maintenance and regulation of neddylation pathway 216, 217. Biologically, DCN1, a scaffold-like co-E3 ligase, promotes the formation of multi-protein complex, while UBC12 facilitates cullin neddylation by recruiting DCN1. Targeting DCN1-UBC12 interaction could selectively regulate cullin neddylation. Structurally, the interaction is mainly mediated by a well-defined binding groove in DCN1 and an N-terminal 12-residue of UBC12 peptide. The groove in DCN1 shows four hydrophobic hotspots, including P1, P2, P3 and P4, which cover those regions occupied by Met1, Ile2 and Leu4 residues of UBC12 separately (Figure 17A-B). These hotspots appear to be amenable for the design of DCN1-UBC12 interaction inhibitors 218.

Based on the observed DCN1-UBC12 interaction, Wang's group determined the 12-residue UBC12 peptide **33** binding to DCN1 with the K_i_ value of 2.6 µM (Figure 17D). Next, they tried to shorten the peptide **33**, leading to hit **34** (K_i_= 50 µM) with a smaller molecular weight (MW) for further optimizations 218. Eventually, compound **35** (DI-591) was provided with the K_i_ value of 12.4 nM and MW of 586, and had excellent aqueous solubility. Compound **35** displayed potent binding affinity to DCN1 and 2 with the K_d_ values of 21.9, 11.2 nM separately, but no inhibitory activity against DCN3-5 at concentration up to 10 µM. The co-crystal structure shows that molecule **35** binds to DCN1 protein with similar binding modes with UBC12 (Figure 17B-C). The bicyclic aromatic group of compound **35** is located in a deep hydrophobic pocket surrounded by I86, F89, C90, F109, F117, A106, A111 and F164. The methyl group of propionyl group in analogue **35** fits well in the hydrophobic pocket defined by L103, L184, Y181, A106 and V102. Additionally, the propionyl group makes hydrogen bond interactions with P97 and Y181. Meanwhile, the amide in the soluble portion of derivative **35** forms a hydrogen bond with Q114. Besides, the cyclohexyl group is half buried in a hydrophobic pocket surrounded by M177, A180, Y181 and L184. Mechanism study indicated that compound **35** could selectively inhibit neddylation of Cul3, resulting in the accumulation of NRF2 protein. In contrast to MLN4924, compound **35** did not exert significantly antiproliferative activity.

**Figure 18:**
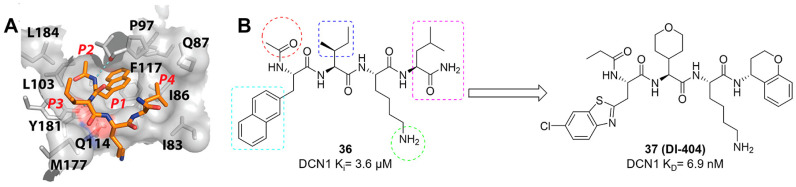
(A) Predicted binding modes of derivative **36** with DCN1. (B) The chemical structures of peptidomimetic compounds** 36**-**37** 219. Reproduced with permission 219. Copyright 2018, American Chemical Society.

As a parallel work to develop compound **35** (DI-591), tetrapeptide **36** (K_i_= 3.6 µM) also was used as a starting point for in-depth optimizations based on the predicted binding modes (Figure 18A), aiming to better target the four pockets (P1, P2, P3 and P4) of DCN1 protein 219. The structural modifications were mainly centered on the marked 5 positions in compound **36**, further producing molecule **37** with the K_D_ value of 6.9 nM (Figure 18B). Compound **37** had an aqueous solubility of 54 μM in PBS at pH = 7.4. In addition, molecule **37** could target DCN1 protein and inhibit DCN1-UBC12 interaction in H2170 cells. Similar to DI-591, derivative **37** selectively suppressed neddylation of cullin 3, causing the accumulation of NRF2 protein, a substrate of CRL3 in H2170 and SK-MES-1 cells.

**Figure 19:**
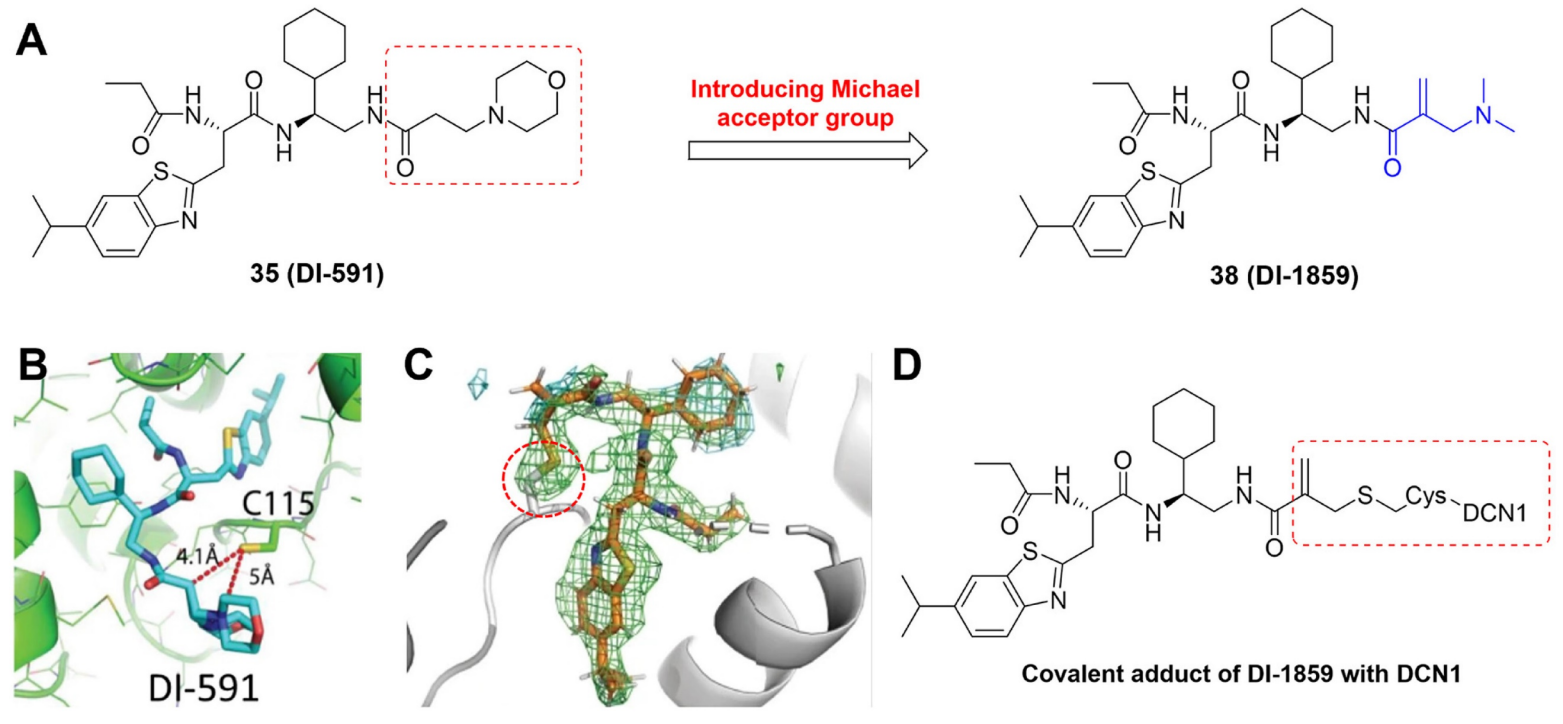
(A) The chemical structures and design strategy of covalent inhibitor **38**. (B) and (C) The co-crystal structures of DCN1 complexed with peptidomimetic compounds **35** and **38** (PDB: 6XOO), respectively. (D) The covalent adduct of peptidomimetic compound **38** with DCN1 142. Reproduced with permission 142. Copyright 2021, Nature Publishing Group.

By analyzing the co-crystal structure of DI-591 complexed with DCN1, Wang's group found that the sulfur atom of Cys115 in DCN1 was located 4.1 Å from the α carbon of the 3-morpholinopropanamide moiety in DI-591 (Figure 19B), which might be a chance for designing covalent DCN1 inhibitors 142. Therefore, a novel class of DI-591 analogues containing various Michael acceptor groups were synthesized and evaluated toward the inhibition of cullin 3 neddylation. In particular, compound **38** (Figure 19A) selectively suppressed cullin 3 neddylation at concentrations of 0.3 nM, about 1000 times stronger than DI-591 in four tested cancer cell lines. From the mass spectrometry analysis, 100% of DCN1 protein was observed to form a complex with compound **38** within 10 min incubation. The co-crystal structure shows that the electron density between Cys115 and molecule **38** verifies the covalent nature of this complex, and the dimethylamino group in molecule **38** is absent (Figure 19C-D). Molecule **38** could specifically target DCN1 over DCN3, although DCN3 has a Cys140 in its binding site corresponding to Cys115 in DCN1. More importantly, they found that pretreated with compound **38** could effectively protect mice from acetaminophen-induced liver damage.

**Figure 20:**
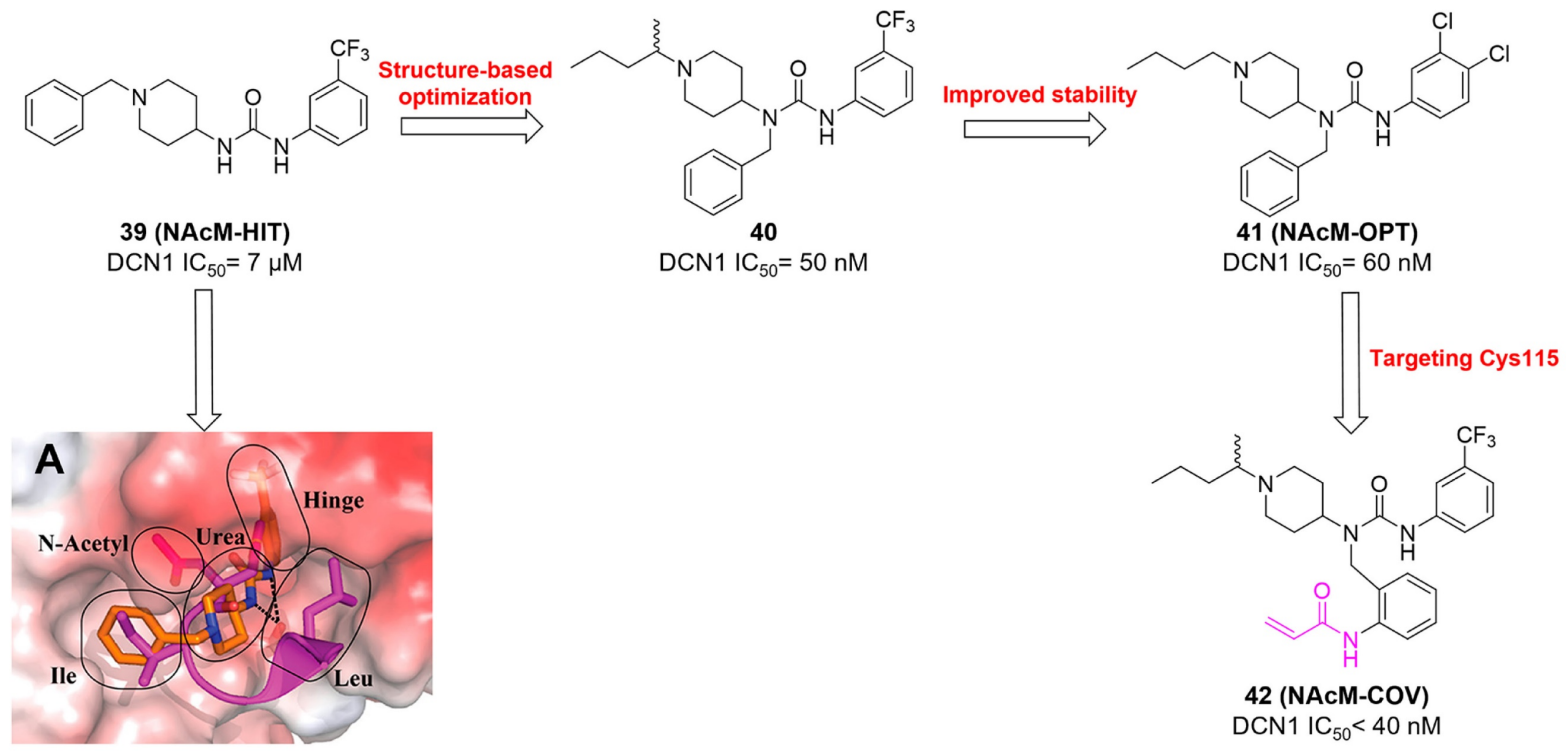
The chemical structures and design strategy of piperidinyl urea derivatives **39**-**42**. (A) The co-crystal structure overlay of **39** (orange) with DCN1 and UBC12^NAc^ (magenta) with DCN1 (PDB: 5V83 and 3TDU), indicating the critical regions targeted for optimizations 220-222. Reproduced with permission 221. Copyright 2018, American Chemical Society.

In 2017, Guy's group reported the screening of a library containing 601,194 unique small molecules using TR-FRET assay, leading to the piperidinyl urea derivative **39** (NAcM-HIT) as a potential DCN1 inhibitor with the IC_50_ value of 7 μM (Figure 20) 220. After analyzing the co-crystal structure, they found that the N-acetyl and Leu pockets in DCN1 were not occupied by compound **39**, which was regarded as an approach to improve the binding affinity (Figure 20A). Thus, the structural optimizations were performed to target the N-acetyl and Leu pockets of DCN1, yielding molecule **40** (Figure 20) with the IC_50_ value of 50 nM, over 100 folds more active than compound **39**. Besides, analogue **40** displayed better solubility and permeability 221. Unfortunately, compound **40** was quickly metabolized in microsomal models (CL_int_= 170 mL/min/kg). Therefore, their continuing optimization efforts were centered on improving the clearance of molecule **40**, through (1) analyzing the physical drivers for bioavailability, and (2) determining metabolic and toxic liabilities related to the scaffold. Subsequently, compound **41** (NAcM-OPT) was provided (Figure 20), which not only possessed favorable biochemical activity (IC_50_= 60 nM), but was significantly more stable (CL_int_= 25 mL/min/kg). Also, derivative **41** was orally bioavailable and well tolerated in mice 222. Based on the co-crystal structure compound **41** with DCN1, Guy and co-authors then developed a covalent inhibitor **42** (NAcM-COV) with more potent binding affinity, whose allylamide group could form a covalent bond with Cys115 of DCN1 (Figure 20). Both compounds **41** and **42** could effectively interrupt DCN1-UBC12 interaction, leading to the deceased neddylation of cullin-1/3 in DCN1-overexpressed HCC95 cells, and inhibited anchorage-independent growth of HCC95 cells 220.

**Figure 21:**
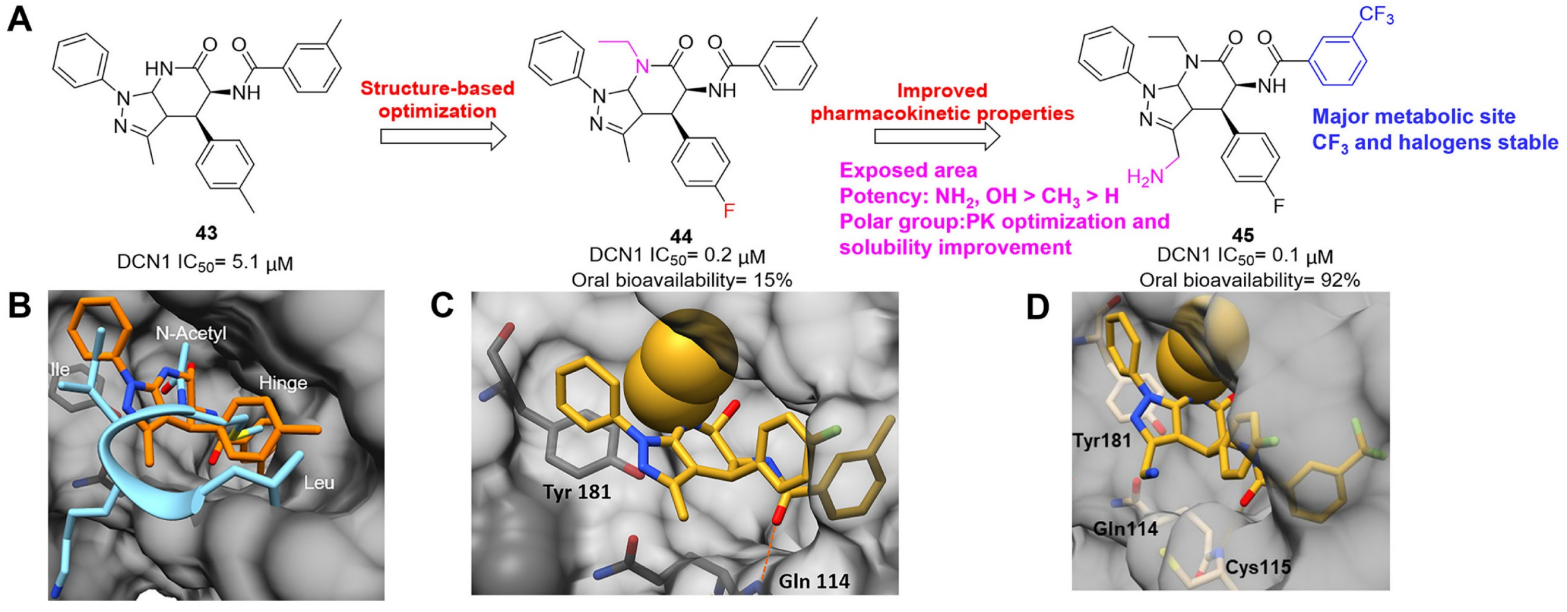
(A) The chemical structures and design strategy of pyrazolo-pyridone derivatives **43**-**45**. (B) The co-crystal structure overlay of **43** (orange) with DCN1 and UBC12^NAc^ (blue) with DCN1 (PDB: 6P5W and 3TDU). Reproduced with permission 223. Copyright 2019, American Chemical Society. (C) The co-crystal structure of **44** with DCN1 (PDB: 6P5V). Reproduced with permission 223. Copyright 2019, American Chemical Society. (D) The co-crystal structure of **45** with DCN1 (PDB: 7KWA) 223, 224. Reproduced with permission 224. Copyright 2021, American Chemical Society.

From the above high-throughput screening results, Guy and co-workers also discovered a pyrazolo-pyridone derivative **43** (Figure 21A), which targeted DCN1-UBC12 interaction with the IC_50_ value of 5.1 μM 223. Compared with the piperidinyl urea derivatives, such as NAcM-OPT, these pyrazolo-pyridone compounds have a high degree of three-dimensional structure that could be beneficial for increasing activity, selectivity and solubility. The co-crystal structure indicates that molecule **43** positions the central pyrazolo-pyridone ring closer to the N-acetyl pocket, which offers a chance for targeting the N-acetyl pocket (Figure 21B). Therefore, the structure-based optimizations were carried out *via* firstly defining the core pharmacophore, followed by identifying the optimal substituents to occupy the key subpockets. Subsequently, analogue **44** (Figure 21A) was generated and showed about 25 folds more potent activity relative to the initial compound **43**. The co-crystal structure of **44** with DCN1 displays that the ethyl group on pyrazolo-pyridone ring well occupies the N-acetyl pocket and pushes the ligand deeper into the Leu pocket (Figure 21C), which is mainly responsible for the improved potency. Considering that compound **44** possessed poor oral bioavailability and relatively rapid clearance, Guy et al. performed further medicinal chemistry optimizations to enhance its bioavailability, yielding derivative **45** (Figure 21A) with the IC_50_ value of 0.1 μM, more potent than **44** 224. Derivative **45** revealed increased aqueous solubility (71 μM), oxidative metabolic stability (MLM Cl_int_< 10 mL/(min kg)), oral bioavailability (92%) and maintained minimal clearance (plasma Cl_IV_ = 1.2 L/(kg h)). The SARs showed that replacement of the methyl pyrazole in **44** with ionizable polar substituents significantly improved aqueous solubility without undesirably influencing the activity. The introduction of CF_3_ group resulted in the electronic and steric deactivation of aryl ring, thus inhibiting oxidative metabolism in microsomal and murine model. Compound **45** possessed stronger thermal stabilization of DCN1 protein in comparison to **44**. Additionally, molecule **45** evidently suppressed anchorage-independent colony formation of HCC95 cells, indicating the main expected phenotypic cellular response. In summary, derivative **45** with potent activity and good oral bioavailability, could be used for exploring the acute pharmacologic effects of DCN1-UBC12 interaction in murine models.

**Figure 22:**
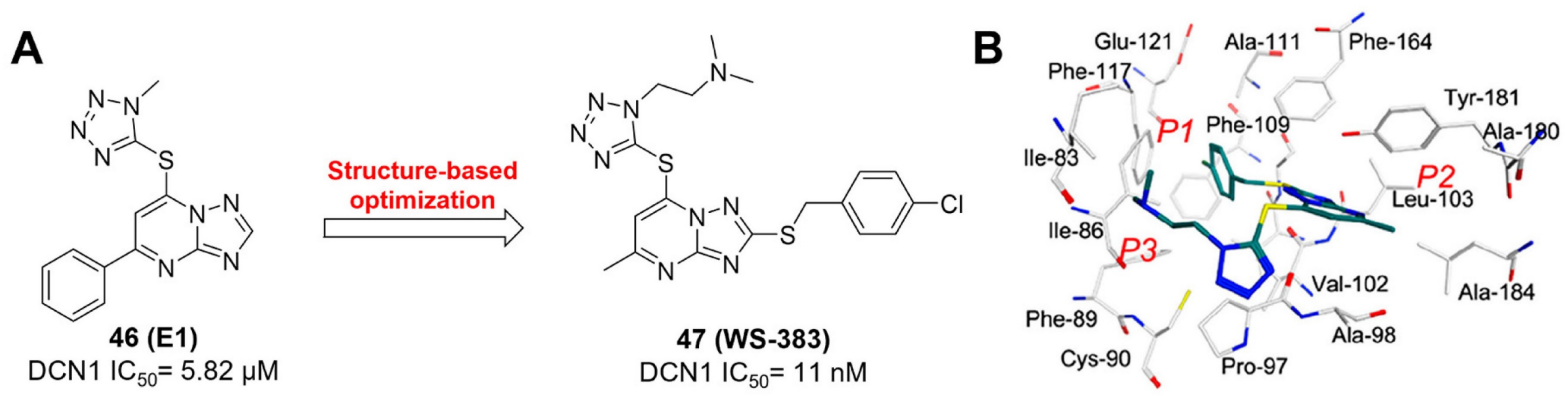
(A) The chemical structures and design strategy of triazolo[1,5-a]pyrimidine derivatives **46**-**47**. (B) The predicted binding modes of compound **47** with DCN1 225. Reproduced with permission 225. Copyright 2019, American Chemical Society.

To find hit compounds targeting DCN1-UBC12 interaction, Wang et al. screened ∼15 000 molecules with 5000 diverse scaffolds using HTRF assay, providing the triazolo[1,5-a]pyrimidine-based hit **46** with the IC_50_ value of 5.82 μM (Figure 22A) 225. Next, structure-based optimizations were carried out based on hit **46**, producing a novel class of triazolo[1,5-a]pyrimidine derivatives, of which compound **47** revealed the strongest binding affinity toward DCN1 protein (IC_50_= 11 nM) in a reversible manner and possessed high selectivity on DCN1 over BTK, CDK and EGFR kinases. The predicted binding modes of **47** with DCN1 shows that the 4-Cl phenyl group is deeply inserted into the hydrophobic pocket, defined by Ile86, Phe89, Val102, Ile105, Ala106, Phe109, Ala111, Phe117 and Phe164, and has π-π stacking interactions with Phe89, Phe109, Phe117 and Phe164 separately (Figure 22B). The 5-methylpyrimidine group is located in the hydrophobic pocket, defined by Ala180, Tyr181 and Leu184. Additionally, the pyrimidine ring possesses an arene-H electrostatic interaction with Ala98. Pro97 has an arene-H electrostatic interaction with the tetrazole ring. Besides, the *N*,*N*-dimethyl group of **47** is observed to form hydrophobic interactions with pocket surrounded by Ile83 and Ile86. These interactions may be responsible for the efficacy of compound **47**. In MGC-803 and KYSE70 cells, molecule **47** selectively suppressed the neddylation of cullin-1/3 over other cullin members, accompanied by the accumulation of their downstream proteins p21, p27 and NRF2. Unfortunately, derivative **47** was metabolized rapidly (T_1/2_< 5 min) and possessed high clearance in human, rat and dog liver microsomes.

**Figure 23:**
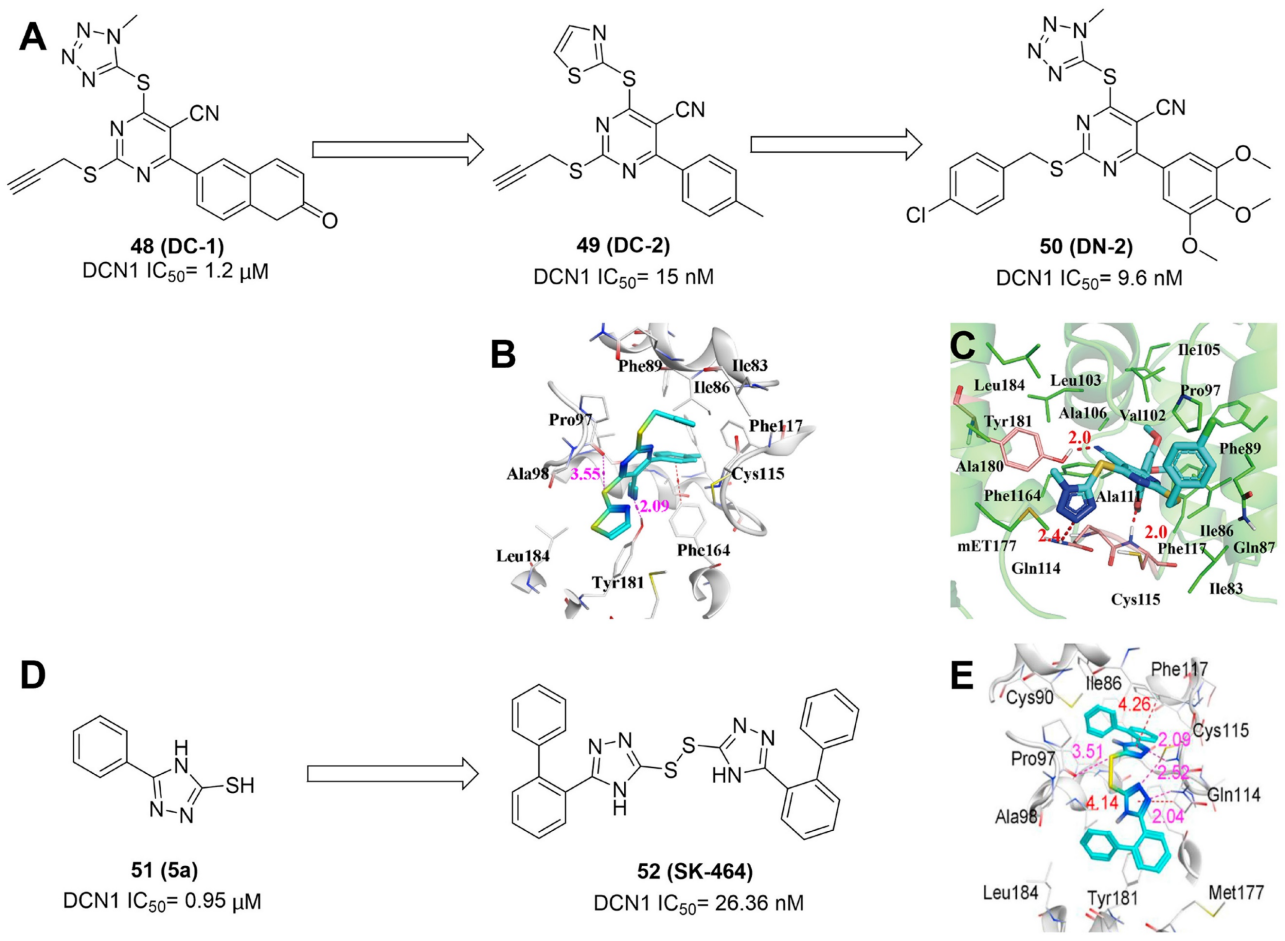
(A and D) The chemical structures of DCN1 inhibitors **48**-**52**. (B) The predicted binding modes of 5‑cyano-6-phenyl-pyrimidine derivative **49** (blue) with DCN1 protein (white) 226. Reproduced with permission 226. Copyright 2019, American Chemical Society. (C) The predicted binding modes of compound **50** (blue) with DCN1 protein (green) 145. Reproduced with permission 145. Copyright 2022, American Chemical Society. (E) The predicted binding modes of compound **52** (blue) with DCN1 protein (white) 227. Reproduced with permission 227. Copyright 2021, Elsevier Masson SAS.

Meanwhile, our group designed and synthesized a novel series of 5‑cyano-6-phenyl-pyrimidine derivatives based on the hit **48** (IC_50_= 1.2 μM) that was identified *via* screening our in-house structurally different molecular library (ca. 1000 compounds), using both FP and HTRF assays (Figure 23A) 226. Among synthesized compounds, molecule **49** disclosed the most potent activity against DCN1-UBC12 interaction with the IC_50_ value of 15 nM, about 80 folds more effective than **48**. Besides,** 49** revealed high selectivity on DCN1-2 over DCN3-5. The docking modes indicate that the sulfur atom connecting the thiazole ring makes a hydrogen bond interaction with the carbonyl group of Pro97 with a distance of 3.55 Å (Figure 23B). The nitrogen atom of cyan group makes a hydrogen bond with the hydroxyl group of Tyr181 with a distance of 2.09 Å. Moreover, the methylbenzene group is extended to the hydrophobic pocket defined by Ile86, Phe89, Val102, Leu103, Ala106 and Phe117, and has an additional arene-H interaction with Phe164. The propargyl group in compound **49** is located in the hydrophobic region surrounded by Ile83, Ile86, Gln87 and Cys115. Preliminary mechanism research indicated that derivative **49** specifically suppressed DCN1-UBC12 interaction, causing the reduction of neddylation of cullin-3 and the accumulation of NRF2 as well as its downstream proteins HO1 and NQO1 in H1975 and PC9 cells.

Based on the docking modes of DCN1 inhibitor **49**, we conducted further structural optimizations and SARs using several drug design strategies, including structure-based drug design and bioisosterism etc., yielding 77 novel 2-thiopyrimidine derivatives as DCN1 inhibitors 145. In comparison to molecule **49**, the new synthesized compound **50** (IC_50_ = 9.55 ± 0.98 nM) possessed stronger inhibitory effect, higher selectivity toward DCN-like proteins and weaker toxicity against cardiac fibroblasts (CFs) (Figure 23A). Molecular docking (Figure 23C), biolayer interferometry, NAE and co-immunoprecipitation assays confirmed that compound **50** could specifically target DCN1 and interfere DCN1-UBC12 interaction. More importantly, we, for the first time, reported that compound **50** could effectively reduce Ang Ⅱ-induced CFs activation, suggesting the potential anti-cardiac fibrotic effect through targeting DCN1.

In addition to hit **48**, we also found the phenyltriazole thiol-based hit **51** as a potential DCN1 inhibitor with the IC_50_ value of 0.95 μM (Figure 23D) 227. Subsequently, structure-based optimizations were performed based on **51**, generating a new array of phenyltriazole thiol analogues. In particular, compound **52** exhibited the strongest biological activity against DCN1-UBC12 interaction with the IC_50_ value of 26.36 nM. In addition, compound **52** disclosed high selectivity and specificity for DCN1 and 2. The predicted binding modes of **52** complexed with DCN1 indicate that in the inside of the pocket, two hydrogen bonds are formed between the nitrogen of triazole ring and the amino group of Cys115, the sulfur of triazole ring and the carbonyl group of Pro97 (Figure 23E). Moreover, the triazole ring makes an arene-H interaction with Ile86, and the diphenyl is extended to the hydrophobic pocked defined by Ile86, Phe89, Cys90, Leu93, Ile105, Ala106, Phe109 and Phe117. In the outside of the pocket, the nitrogen atoms of the triazole make two hydrogen bond interactions with Gln114 and Cys115, respectively. The diphenyl forms hydrophobic interactions with Ala98, Leu103, Met177, Ala180, Tyr181 and Leu184. Further exploration showed that compound **52** could bind to DCN1, block the neddylation of cullin-3 and suppress KYSE70 and H2170 cell migration and invasion.

Taken together, since 2017, DCN1 inhibitors with various chemotypes have been identified, including peptidomimetic derivatives, piperidinyl urea derivatives, pyrazolo-pyridone derivatives, triazolo[1,5-a]pyrimidine derivatives, 5‑cyano-6-phenyl-pyrimidine derivatives and phenyltriazole thiol-based derivatives. These DCN1 inhibitors were found mainly *via* structure-based drug design, high throughput virtual screening and screening in-house structurally different molecular library. The most reported DCN1 inhibitors showed robust binding affinity with the IC_50_ values ranging from 1 to 200 nmol/L. Covalent DCN1 inhibitors seemed to be more potent than non-covalent DCN1 inhibitors. Owing to the high homology between DCN1 and DCN2, all the reported DCN1 inhibitors were highly selective toward DCN1-2 over DCN3-5. Interestingly, the structural differences of DCN1 inhibitors may result in their diverse effects on inhibiting cullin3 or/and cullin1 neddylation. In addition, many DCN1 inhibitors were lack of favorable pharmacokinetic and physicochemical properties. Besides, DCN1 was initially regarded as a promising anti-cancer therapeutical target, while the anti-cancer effects of DCN1 inhibitors were subsequently found to be weak. Considering the NRF2, an antioxidant transcription factor, is a typical substrate of cullin3 10, DCN1 inhibitors could play extensive efficacy for the treatment of diseases linked to over-production of reactive oxygen species (ROS). Especially, the latest findings indicated that DCN1 inhibitors exhibited effective potency on relieving acetaminophen-induced liver damage and AngⅡ -induced cardiac fibrosis, which was related with the inhibition of cullin3 neddylation and its substrate NRF2 activation 142, 145. All in all, more potent and selective DCN1 inhibitors with drug-like properties are urgently required, aiming to further investigate their therapeutical potential for human diseases and DCN1 protein's potentially biological roles.

### Cullin 1/5 inhibitors

**Figure 24:**
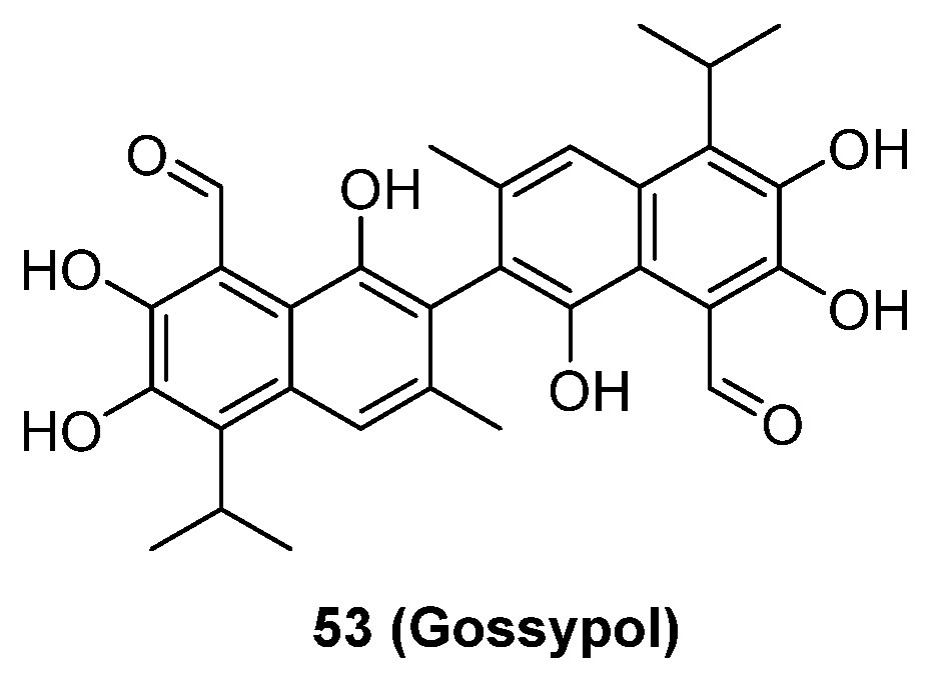
The chemical structure of Cul1/5 inhibitor Gossypol 228.

To discover potent and novel inhibitors targeting Cul5 neddylation, Sun's group screened a library of 17,000 molecules using an AlphaScreen-based assay, yielding the natural **53** (gossypol) as a potent inhibitor of cullin neddylation (Figure 24) 228, 229. Molecule **53** effectively inhibited the neddylation of Cul5 and Cul1 with the IC_50_ values of 4.3 and 3.4 μM, respectively. Besides, it could straightly bind to SAG-Cul5 and RBX1-Cul1 complexes in multiple cancer cells, accompanied by accumulation of Cul5 substrate NOXA and Cul1 substrate MCL1. Interestingly, the combination of compound **53** and MCL1 inhibitor S63845 displayed a synergistic anticancer effect in multiple human cancer cells.

### UBE2F inhibitors

In 2022, Sun's research group, for the first time, reported an azaindole-based UBE2F inhibitor HA-9104, which was obtained through a structure-based virtual screen and multiple rounds of chemistry-based optimization 230. HA-9104 could effectively bind to UBE2F protein and decrease its expression, resulting in the inhibition of cullin-5 neddylation. In addition, the inhibition of cullin-5 neddylation inactivated CRL-5 activity, further causing the accumulation of NOXA, a substrate of CRL-5, to induce apoptosis. Besides, the 7-azaindole group of HA-9104 may form a DNA adduct to cause DNA damage and G2/M arrest. Furthermore, HA-9104 displayed potent antiproliferative activity against lung cancer H1650, H2170, H358 cells with the IC_50_ values of 5.260, 1.765, 4.377 μΜ separately and possessed radiosensitization in both *in vitro* cell culture and *in vivo* xenograft tumor models. Taken together, HA-9104, a novel inhibitor of UBE2F-CRL5 axis, showed highly anti-cancer activity alone or in combination with radiation, and remained to be needed further optimization and development.

### UBC12 inhibitors

In 2022, Chen and co-authors, for the first time, disclosed an UBC12 inhibitor arctigenin *via* screening a natural product library with 32 compounds 231. Arctigenin could significantly decrease the level of NEDD8-conjugated UBC12, further resulting in the reduction of the neddylation levels of cullin 1, cullin 3 and cullin 4. Additionally, treated with arctigenin dose-dependently suppressed the growth of UBC12 overexpressed PC9, H1299 and HGC27 cells with IC_50_ values of 9.7, 17.2 and 9.5 μM separately. Further mechanism studies indicated that arctigenin obviously inhibited cullin neddylation in gastric cancer cells, causing the accumulation of cullin downstream substrate PDCD4 that was a tumor suppressive protein and could induce cell death. Besides, the suppression of UBC12 and cullin neddylation was beneficial for regulating protein stability by arctigenin.

## Conclusions and perspectives

The overactivated neddylation proteins and CRL components play pivotal roles on cancer progression and confer poor overall survival, indicating that targeting neddylation is a promising strategy for cancer treatment. However, there is challenging to identify which of neddylation proteins probably possess the strongest therapeutic effects when being targeted, and how to develop effective and selective drugs that can inhibit these proteins without side effects.

NAE is regarded as a promising therapeutic target for human diseases, and its inhibitor MLN4924 is undergoing many phase I/Ⅱ/III trials for cancer treatment. However, treated with MLN4924 in phase I trials unexpectedly led to modest response 152-154, the emergence of resistance owing to heterozygous mutations in UBA3 32, 148, 149, 151, and liver toxicity 152. The discovery of small molecules targeting NAE mutations might be an effective way to reduce resistance. In addition, some NAE inhibitors also displayed significantly inhibitory activity against other E1, like UAE and SAE, which may cause inevitable side effects. Therefore, it was necessary to find the differences between the active sites of these E1 proteins to better design selective NAE inhibitors. Besides, numerous clinical trials and preclinical studies disclosed that MLN4924 could be regarded as a sensitizer in the combination with chemo- or radiotherapy, which exerted stronger efficacy for a variety of chemo-/radio-resistant cancers (Table 2-4). Especially, two phase III trials of MLN4924 in combination with azacytidine have been launched for the treatment of AML, MS and CMML. These findings show that targeting NAE is a meaningful strategy for cancers, and more potent and specific NAE inhibitors with low toxicity are still desired.

In addition to NAE inhibitors, many potent DCN1-UBC12 interaction inhibitors were reported and could selectively inhibit neddylation of cullin 3 and/or cullin 1, leading to more specifically explore the pharmacological roles of individual CRL. Although DCN1 was a promising therapeutical target for the treatment of diseases, DCN1 inhibitors displayed limited anticancer potency *in vitro*. Also, the effects of DCN1 inhibitors at the cellular level remain moderate, indicating more efforts needed to further enhance their cellular efficacy. Of note, compared to non-covalent DCN1 inhibitors like DI-591, covalent DCN1 inhibitors like DI-1859 seemed to have stronger cellular potency, suggesting that the development of covalent DCN1 inhibitors may have more important value 142. Moreover, given that the antioxidant transcription factor NRF2 is a typical substrate of cullin3 10, DCN1 inhibitors probably play vital role for the treatment of diseases related to over-production of ROS. In particular, the recent findings disclosed that DCN1 inhibitors possessed evident potency on reducing acetaminophen-induced liver damage and AngⅡ-induced cardiac fibrosis, connected with the inhibition of cullin3 neddylation and its substrate NRF2 activation 142, 145. Besides, considering that neddylation inhibition well sensitize chemo-/radio-resistant cancer cells 159, 167, 172, DCN1 inhibitors can be used to investigate their potency on relieving resistance in the future. Anyhow, more powerful and selective DCN1 inhibitors with favorable drug-like properties should be developed in the future, aiming to further explore the potential functions of DCN1 protein in human diseases.

Overall, the development of neddylation inhibitors remains in infancy and it is highly desired to develop more active agents selectively targeting neddylation E2s (UBC12 or UBE2F), E3s (such as RBX1 or RBX2) or E3-substrates for cancer treatment. Considering the potential anti-cancer potency of NAE activators 214, 215. the design of modulators activating neddylation proteins may be also a new therapeutical strategy for human diseases. In addition, neddylation protein degradation may be an effective approach for treating diseases caused by neddylation protein mutation or activation. Until now, no structures of neddylation protein in complex with PROTAC-based degraders have been reported, more efforts are needed to solve the crystal structures of these proteins to guide the rational degrader design. The application of highly accurate protein structure prediction technologies, such as alphafold 232, may be an important opportunity to predict the crystal structures unresolved protein in neddylation pathway. Furthermore, trivalent and dual-targeting PROTACs might also have therapeutic potential for treating neddylation dysregulated human diseases.

Finally, we want to highlight the critical roles of targeting neddylation for fibrotic diseases. Although important involvement of neddylation, the therapeutic roles of targeting neddylation pathway or specific enzymes of this pathway in fibrotic diseases remain to be further explored.

## Figures and Tables

**Figure 25:**
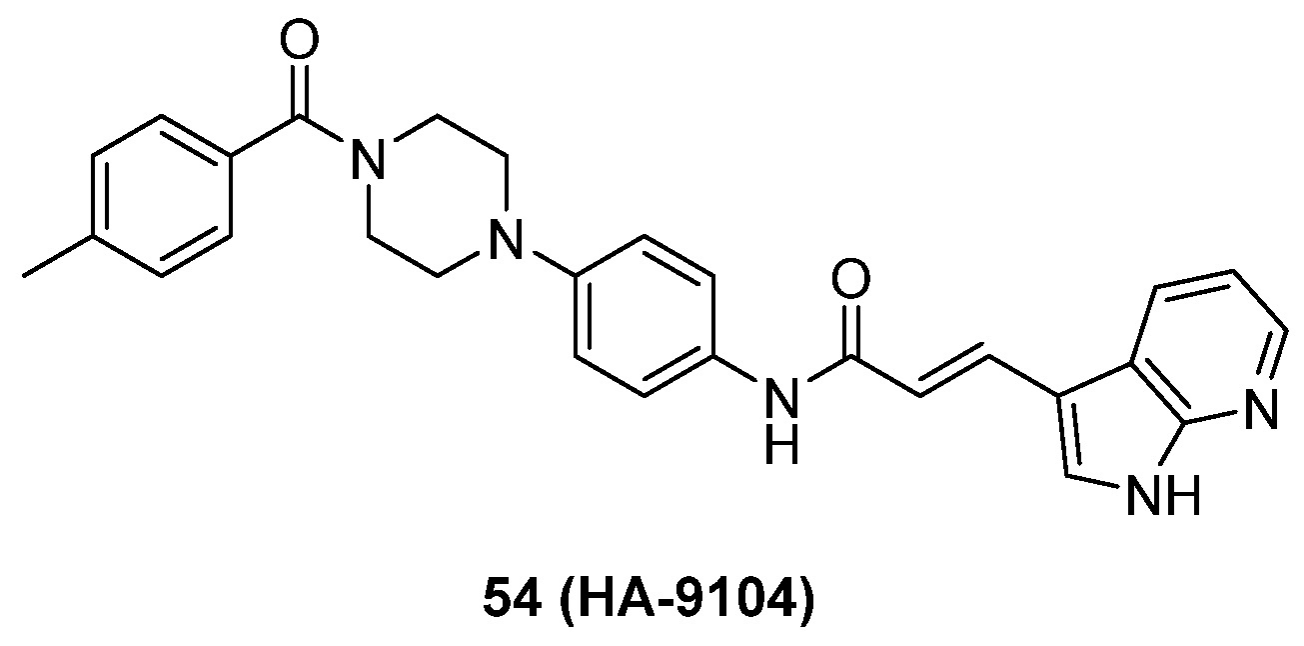
The chemical structure of UBE2F inhibitor HA-9104 230.

**Figure 26:**
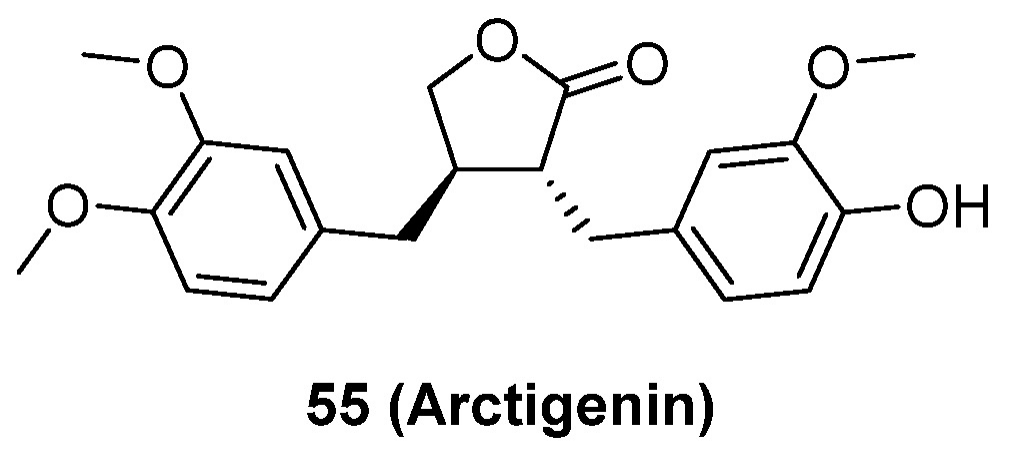
The chemical structure of UBC12 inhibitor arctigenin 231.

**Figure 27:**
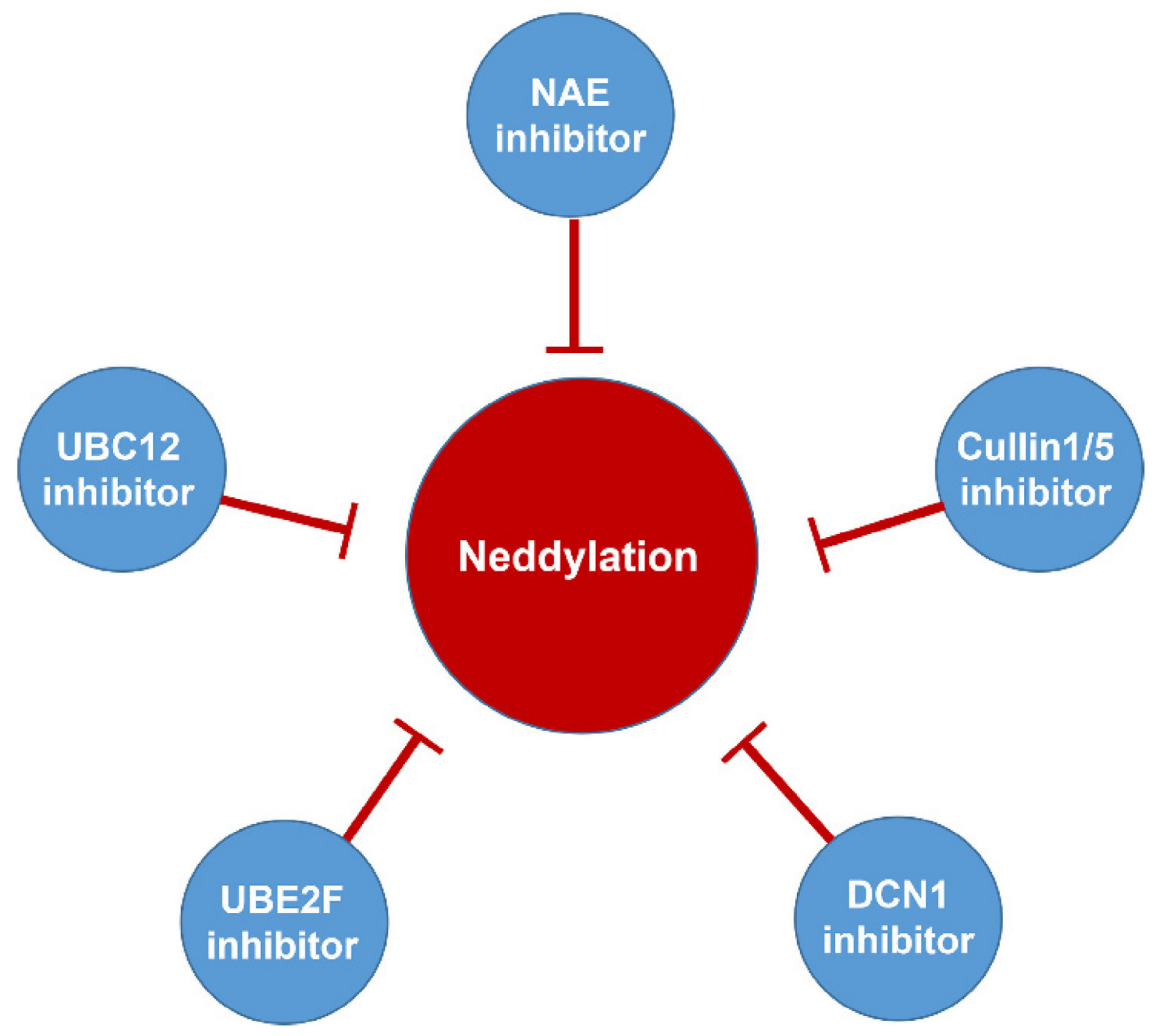
A summary of targeting neddylation through inhibiting various proteins of this pathway.

**Table 1:** The reported neddylation proteins are overactivated in different cancers.

Neddylation proteins	Cancer type
NAE	lung cancer, liver cancer, leukemia, glioblastoma, HNSCC, melanoma, colorectal cancer, prostate cancer, breast cancer, esophageal cancer, gastric cancer, myeloma, lymphoma, ovarian cancer, bladder cancer, chondrosarcoma, cervical carcinoma, pancreatic cancer
NEDD8	lung cancer, liver cancer, leukemia, glioblastoma, HNSCC, colorectal cancer, prostate cancer, breast cancer, esophageal cancer, gastric cancer, bladder cancer, oral squamous cell carcinoma
UBC12	lung cancer, liver cancer, HNSCC, prostate cancer, breast cancer, esophageal cancer, gastric cancer, ovarian cancer
UBE2F	lung cancer, colorectal cancer, ovarian cancer
RBX1	lung cancer, liver cancer, leukemia, melanoma, colorectal cancer, prostate cancer, breast cancer, esophageal cancer, gastric cancer, myeloma, lymphoma, ovarian cancer, bladder cancer
RBX2	lung cancer, leukemia, glioblastoma, colorectal cancer, prostate cancer, ovarian cancer, pancreatic cancer
DCN1	lung cancer, gastric cancer
Cul1	lung cancer, liver cancer, HNSCC, melanoma, colorectal cancer, prostate cancer, breast cancer, gastric cancer
Cul2	glioblastoma
Cul4A	liver cancer, melanoma, ovarian cancer
Cul4B	liver cancer, melanoma
Cul5	glioblastoma, bowel cancer
Cul7	liver cancer

**Table 2:** Clinical trials of MLN4924 tested for cancer treatment.

Identifier	Phase	Cancer type	With combination	Status	Patientnumber	Initial time	Ref.
NCT00677170	I	Solid tumor	Alone	Completed	62	2008,03	152
NCT00722488	I	HM, MM, lymphoma, HL	Alone	Completed	56	2008,07	153
NCT00911066	I	AML, MS, ALL	Azacitidine	Completed	72	2009,03	154
NCT01011530	I	MM	Alone	Completed	37	2009,11	155
NCT01415765	I/II	B-cell lymphoma	Alone	Withdrawn	-	2011,07	NR
NCT01814826	I	AML	Azacitidine	Completed	64	2013,03	156
NCT01862328	I	Solid tumor	Docetaxel, Gemcitabine, Carboplatin, Paclitaxel	Completed	64	2013,05	NR
NCT02122770	I	Solid tumor	Fluconazole, Itraconazole, Docetaxel, Carboplatin, Paclitaxel	Completed	52	2014,04	NR
NCT02782468	I	AML	Azacitidine	Not yet recruiting	37	2016,03	NR
NCT02610777	II	MS, CMML, AML	Azacitidine	Not yet recruiting	120	2016,04	NR
NCT03013998	I/II	AML	Azacitidine	Recruiting	500	2016,11	NR
NCT03057366	I	Solid tumors	Docetaxel, Carboplatin, Paclitaxel	Completed	8	2017,02	NR
NCT03009240	I	AML	Decitabine	Recruiting	30	2017,08	NR
NCT03238248	I	MS, MN	Azacitidine	Recruiting	71	2017,08	NR
NCT03319537	I/II	Mesothelioma	Pemetrexed, Cisplatin	Recruiting	42	2017,10	NR
NCT03323034	I	Neoplasm, lymphoma	Temozolomide, Irinotecan	Recruiting	76	2017,11	NR
NCT03268954	III	MS, CMML, AML	Azacitidine	Recruiting	450	2017,11	NR
NCT03330106	I	Advanced solid neoplasm	Docetaxel, Carboplatin, Paclitaxel	Recruiting	45	2017,11	NR
NCT03228186	I	NSCLS	Docetaxel	Recruiting	37	2018,01	NR
NCT03459859	I	AML, MS	Cytarabine	Recruiting	18	2018,03	NR
NCT03479268	I	CLL, Non-HL	Ibrutinib	Recruiting	30	2018,03	NR
NCT03386214	I	Myelofibrosis	Ruxolitinib	Recruiting	18	2018,04	NR
NCT03330821	I/II	AML	Cytarabine, Idarubicin	Recruiting	53	2018,04	NR
NCT03709576	II	AML	Azacitidine	Recruiting	30	2018,07	NR
NCT03486314	I	Advanced solid neoplasm	Rifampin, Docetaxel, Carboplatin, Paclitaxel	Recruiting	20	2018,08	NR
NCT03814005	I	MS, CMML, AML	Azacitidine	Not yet recruiting	20	2019,02	NR
NCT03862157	I/II	AML	Azacitidine, Venetoclax	Recruiting	40	2019,02	NR
NCT03349281	I	ALL	Vincristine, Dexamethasone, PEGasparaginase, Doxorubicin	Recruiting	18	2019,03	NR
NCT03772925	I	AML, MS	Belinostat	Not yet recruiting	45	2019,03	NR
NCT03745352	I	AML	Azacitidine	Not yet recruiting	72	2019,03	NR
NCT03813147	I	AML, MS	Azacitidine, Fludarabine, phosphate, Cytarabine	Not yet recruiting	23	2019,05	NR
NCT03770260	I	Multiple myeloma	Ixazomib citrate	Not yet recruiting	54	2019,07	NR
NCT04090736	III	AML	Azacitidine	Recruiting	466	2019,08	NR
NCT03965689	II	NSCLC	Carboplatin, Paclitaxel	Recruiting	25	2019,09	NR
NCT04175912	II	Advanced intrahepatic cholangiocarcinoma	Carboplatin, Paclitaxel	Suspended	52	2020,01	NR
NCT04172844	I	AML	Azacitidine, Venetoclax	Recruiting	24	2020,01	NR
NCT04266795	II	AML	Venetoclax, Azacitidine	Recruiting	150	2020,10	NR
NCT04712942	II	MDS, AML	Azacitidine	Recruiting	102	2021,01	NR
NCT04800627	I/II	dMMR/MSI-H cancers	Pembrolizumab	Recruiting	39	2021,03	NR
NCT04484363	-	Myelodysplastic syndromes	Azacitidine	Available	-	-	NR

NR, not reported

**Table 3:** MLN4924 serves as a chemosensitizer.

Drug	Cancer type	Mechanisms	Ref.
Cisplatin	Colon cancer	(1) Suppression of DNA damage-induced FANCD2 monoubiquitination and CHK1 phosphorylation. (2) Increases SUB1/PC4 expression in response to DNA damage, oxidative stress and apoptosis.	157, 158
	Ovarian cancer	(1) Silencing of Cul3. (2) Promotes DNA damage, oxidative stress and expression of the BH3-only protein NBK/BIK.	159, 160
	Urothelial Carcinoma,	Promotes DNA damage, JNK activation and down-regulation of Bcl-Xl	161
	Cervical carcinoma,	NR	162
	Esophageal cancer	Induces apoptosis	163
	Pancreatic cancer	Promotes DNA damage and the expression of apoptosis-associated proteins through enhancing CDT1, ORC1, p27, p-IκBα and p21 accumulation.	164
	Triple-negative breast cancer	Increases the DNA damage	165
	Diffuse large B-cell lymphoma	NR	166
Carboplatin	Ovarian cancer	NR	167
Azacitidine	AML	Inhibits RRM2 expression and induces apoptosis	168
Cytarabine	AML	(1) MLN4924 promotes cytarabine into the DNA of AML cells. (2) Increases DNA damage.	169
Belinostat	AML	Disables DNA damage response	170, 171
All-trans retinoic acid	AML	Induction of apoptosis is related to accumulation of pro-apoptotic proteins, NOXA and c-JUN.	172
Mitomycin C	Colon cancer, melanoma, lung cancer, osteosarcoma	Induces cell death	173
Bortezomib	MM	Induces apoptosis	127
Bendamustine, chlorambucil	CLL	Induces DNA damage, checkpoint activation, cell cycle arrest and apoptosis.	174
Olaparib	NSCLS	Impairs the DNA repair process	175
JQ1	Pancreatic adenocarcinoma	Induces ROS that promotes the increased DNA damage, causing apoptosis.	176
Gemcitabine	Pancreatic ductal adenocarcinoma	Accumulation of the pro-apoptotic protein NOXA	177
MK-2206	Breast cancer	Induces apoptosis	178
Imatinib	Leukemia	Induces DNA damage and triggers a dramatic shift in the expression of MCL1 and NOXA	179
Fulvestrant	Breast cancer	Targeting neddylation with MLN4924 can transcriptionally inhibit ER-α expression through SGK1-dependent nuclear export of FOXO3a.	116
Sorafenib	Hepatocellular carcinoma	Upregulation of CRL/Skp1-Cullin1-F-box E3 ubiquitin ligase substrates p21, p27, Deptor and IκBɑ	180
Tamoxifen	Breast cancer	Inactivates the FBXW2-MSX2-SOX2 axis	181

**Table 4:** MLN4924 serves as a radiosensitizer.

Cancer type	Mechanisms	Ref.
Pancreatic cancer	Induces accumulation of CDT1 and WEE1 to trigger DNA damage response and G2/M arrest	182
Breast cancer	Induction of p21 and apoptosis-related gene, caspase-3	183, 184
Colorectal cancer	Accumulation of p27	185
Prostate cancer	(1) Accumulation of WEE1/p21/p27. (2) Induction of DNA damage and apoptosis.	186
Head and neck squamous carcinoma	Induction of re-replication	97

**Table 5:** A summary of the reported NAE modulator.

Compound ID	Structure	Year reported	Biological functions	Reference
**1** (MLN4924)	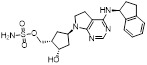	2009	A selective and covalent NAE inhibitor (IC_50_= 4 nM) The detailed biological functions of MLN4924 in cancers were introduced in the part 3 of this manuscript.	30
**2** (compound 1)	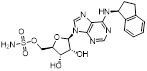	2010	An equipotent inhibitor of NAE, UAE, and SAE-dependent transthiolation reactions with IC_50_ values of 5 nM	147, 150
**3**	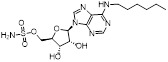	2011	A selective and covalent NAE inhibitor (IC_50_ < 10 nM) Potent antiproliferative activity toward K562 leukemia cells (IC_50_= 160 nM)	187
**4** (ABP1)	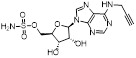	2013	ABPQ, a nonselective and covalent E1 inhibitor, could decrease the expression levels of Ub, NEDD8, SUMO1/2/3 and Ufm1 conjugates in A549 cells.	188
**5** (ABP A3)	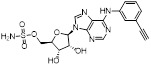	2015	ABP A3, a dual inhibitor of Ub and NAE inhibitor, possessed potent anti-cancer activity toward A549 cells (IC_50_= 2.5 μM), and could induce A549 cell apoptosis.	189
**6** (TAS4464)	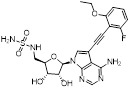	2019	A selective and covalent NAE inhibitor (IC_50_= 0.955 nM) TAS4464 showed extensively anticancer activity, and revealed a wider therapeutic index than MLN4924. However, owing to the potential hepatic and gastrointestinal toxicity in the phase 1 study, TAS4464 was terminated in the subsequent clinical evaluation.	190, 192, 193
**7**	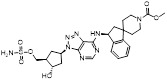	2021	A potent NAE and UAE dual inhibitor with the IC_50_ values of 0.55, 66.84 nM separately. molecule 7 revealed potent antitumor potency and a good safety profile in MV-4-11 and HCT-116 xenograft models.	191
**8** (LZ3)	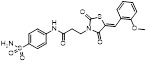	2014	A covalent NAE inhibitor (IC_50_= 1.06 μM), Caco-2 cells (IC_50_= 12.3 μM)	194
**9** (LZ6)	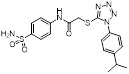	2014	A covalent NAE inhibitor (IC_50_= 9.18 μM)	194
**10** (LZ7)	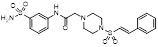	2014	A covalent NAE inhibitor (IC_50_= 19.71 μM)	194
**11** (6,6''-biapigenin)	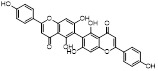	2011	A non-covalent NAE inhibitor (IC_50_= 20 μM), Caco-2 cells (IC_50_= 25 μM)	195
**12-1**	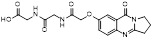	2012	A selective and non-covalent NAE inhibitor (IC_50_= 0.8 μM), Caco-2 cells (IC_50_= 10 μM)	196
**12-2**	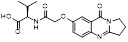	2015	A selective and non-covalent NAE inhibitor (IC_50_= 0.39 μM)	197
**12-3**	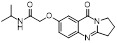	2015	A selective and non-covalent NAE inhibitor (IC_50_= 0.27 μM)	197
13 (Flavokawain A)	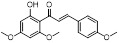	2015	A non-covalent NAE inhibitor (IC_50_= 5 μM) Flavokawain A inhibited the formation of high-grade prostatic intra-epithelial neoplasia lesions and prostate adenocarcinomas.	199
**14** (Flavokawain B)	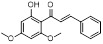	2019	A non-covalent NAE inhibitor (IC_50_= 11 μM) Flavokawain B's inhibitory activity toward prostate cancer cells is owing to FKB's binding to the NAE regulatory subunit APP-BP1.	198
**15** (Gartanin)	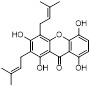	2019	A non-covalent NAE inhibitor (IC_50_= 0.735 μM) Gartanin effectively inhibited the growth of prostate cancer lines through autophagy initiation.	200
**16**	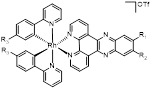	2012	A non-covalent NAE inhibitor (IC_50_= 1.5 μM), Caco-2 cells (IC_50_= 0.3 μM) Compound 16 suppressed CRL substrate degradation and NF-kB activation in Caco-2 cells.	201
**17**	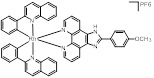	2016	A non-covalent NAE inhibitor (IC_50_= 0.1 μM), Caco-2 cells (IC_50_= 4.3 μM) Complex 17 indicated promising effect on the treatment of inflammatory bowel disease.	202
**18** (Piperacillin)	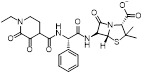	2014	A selective and non-covalent NAE inhibitor (IC_50_= 1 μM) Compound 18 could effectively inhibit the degradation of NAE downstream protein substrate p27^kip1^.	203
**19** (Mitoxantrone)	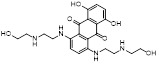	2018	A selective and non-covalent NAE inhibitor (IC_50_= 1 μM) Analogue 19 could induce Caco-2 cell apoptosis through regulation of cullin-dependent substrates p53.	204
**20** (Candesartan cilexetic)	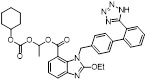	2020	A non-covalent NAE inhibitor (IC_50_= 16.43 μM), A549 cells (IC_50_= 63.93 μM) Compound 20-treated with 60 or 30 mg/kg group effectively inhibited the growth of A549 xenograft tumors.	205
**21**	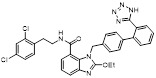	2021	A non-covalent NAE inhibitor (IC_50_= 5.51 μM), A549 cells (Inhibition rate= 83.9%, 100μM) Compound 21 not only could induce A549 cell apoptosis and senescence, but also inhibit the growth of A549 xenograft tumors.	206
**23** (LP0040)	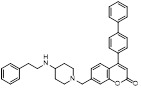	2018	A NAE/UAE dual inhibitor (IC_50_= 0.37 μM), AGS cells (IC_50_= 0.76 μM) Compound 23 effectively induced AGS cell G1 phase arrest and apoptosis.	208
**24**	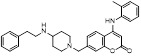	2020	A non-covalent NAE inhibitor (IC_50_= 0.56 μM), BxPC-3 cells (IC_50_= 0.17 μM) Derivative 24 induced BxPC-3 cell apoptosis and played a synergistic effect with bortezomib on BxPC-3 cell growth inhibition.	209
**26** (ZM223)	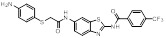	2017	A non-covalent NAE inhibitor, HCT116 cells (IC_50_= 0.1 μM) U-2OS cells (IC_50_= 1.22 μM)	210
**28** (V11)	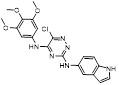	2020	Compound 28 evidently inhibited the activity of NAE in MGC803 cells (EC_50_= 3.56 μM). Molecule 28 displayed modest anticancer activity toward MGC803, PC3, MCF-7 cells with the IC_50_ values of 8.22, 9.28, 9.64 μM separately.	211
**29** (V7)	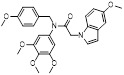	2021	A non-covalent NAE inhibitor Compound 29 exhibited potent biological activity toward MGC803, PC3 and EC109 cells with the IC_50_ values of 1.59, 3.56, 14.52 μM. Induction of MGC803 cell G2/M phase arrest and apoptosis	212
**30** (HA-1141)	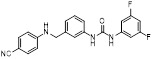	2021	A non-covalent NAE inhibitor Compound 30 possessed the dual activities of blocking neddylation and triggering ER stress, leading to growth suppression of cancer cells.	213
**31**	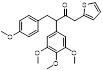	2020	An NAE activator Induction of MGC803 cell G2/M phase arrest and apoptosis Compound 31 could activate NAE1-UBC12-Cullin1 neddylation through binding NAE straightly, leading to the degradation of anti-apoptosis protein IAPs.	214
**32** (K3)	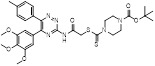	2021	An NAE activator Compound 32 displayed potent cytotoxicity on MGC-803, PC-3 and EC-109 cell lines with the IC_50_ values of 2.35, 5.71 and 10.1 μM, separately. Induction of MGC-803 and HGC-27 cell growth inhibition and apoptosis	215

**Table 6:** A summary of the reported DCN1 inhibitors.

Inhibitors	Year reported	DCN1 (IC_50_)	Half-life	Biology	Reference
**35** (DI-591)	2017	12 nmol/L	-	A non-covalent DCN1 inhibitor High selectivity toward DCN1-2 over DCN3-5 Selective inhibition of cullin 3 neddylation	218
**37** (DI-404)	2018	6.9 nmol/L	-	A non-covalent DCN1 inhibitor Selective inhibition of cullin 3 neddylation	219
**38** (DI-1859)	2021	< 1 nmol/L	-	A covalent DCN1 inhibitor High selectivity toward DCN1-2 over DCN3-5 Compound 38 selectively suppressed cullin 3 neddylation at concentrations of 0.3 nM, about 1000 times stronger than DI-591. Also, compound 38 effectively protected mice from acetaminophen-induced liver damage.	142
**40** (7)	2018	50 nmol/L	iv: 0.14 h	A non-covalent DCN1 inhibitor High selectivity toward DCN1-2 over DCN3-5 Compound 40 not only had favorable solubility and permeability, but also could selectively reduce steady-state levels of neddylated cullin1 and cullin3.	222
**41** (OPT)	2018	60 nmol/L	iv: 3.6 h, po: 4.2 h	A non-covalent DCN1 inhibitor High selectivity toward DCN1-2 over DCN3-5 Selective inhibition of cullin 1 and cullin 3 neddylation	220-222
**42** (COV)	2018	< 40 nmol/L	-	A covalent DCN1 inhibitor High selectivity toward DCN1-2 over DCN3-5 Selective inhibition of cullin 1 and cullin 3 neddylation	220
**44** (27)	2019	200 nmol/L	iv: 1.2 h	A non-covalent DCN1 inhibitor High selectivity toward DCN1-2 over DCN3-5 Selective inhibition of cullin 1 and cullin 3 neddylation	223
**45** (40)	2021	100 nmol/L	iv: 7.2 h, po: 9.5 h	A non-covalent DCN1 inhibitor High selectivity toward DCN1-2 over DCN3-5 Selective inhibition of cullin 1 and cullin 3 neddylation	224
**47** (WS-383)	2019	11 nmol/L	< 5 min	A non-covalent DCN1 inhibitor Selective inhibition of cullin 1 and cullin 3 neddylation	225
**49** (DC-2)	2019	15 nmol/L	< 5 min	A non-covalent DCN1 inhibitor High selectivity toward DCN1-2 over DCN3-5 Selective inhibition of cullin 3 neddylation	226
**50** (DN-2)	2022	9.55 nmol/L	< 5 min	A non-covalent DCN1 inhibitor High selectivity toward DCN1-2 over DCN3-5 Selective inhibition of cullin 3 neddylation Potential anti-cardiac fibrotic effect	145
**52** (SK-464)	2021	26 nmol/L	iv: 0.63 h	A non-covalent DCN1 inhibitor High selectivity toward DCN1-2 over DCN3-5 Compound 52 selectively blocked the neddylation of cullin 3 and suppressed KYSE70 and H2170 cell migration and invasion.	227

## Abbreviations

ABL: Abelson oncogene
AEs: Adverse events
AML: Acute myelogenous leukemia
Ang II: Angiotensin II
AP-1: Activating protein-1
APL: Acute promyelocytic leukemia
APPBP1: Amyloid beta precursor protein binding protein 1
AR: Androgen receptor
AR-Vs: AR and its active variants
ATF4: Activating transcription factor 4
ATP: Adenosine-triphosphate
ATRA: All-trans retinoic acid
BCR: Breakpoint cluster region
BTK: Bruton'styrosinekinase
Cand1: Neddylation-dissociated 1 protein
CC: Cervical cancer
CCL2: Chemotactic cytokine ligand 2
CDK: Cyclin-dependent kinases
CLL: Chronic lymphocytic leukemia
CML: Chronic myeloid leukemia
CRC: Colorectal cancer
CRLs: Cullin-RING ligases
DCN1: Defective in cullin neddylation 1
DLBCL: Diffuse large B-cell lymphoma
DR5: Death receptor 5
EGFR: Epidermal growth factor receptor
Erk: Extracellular regulated protein kinases
ERRβ: Estrogen-related receptor beta
ESCC: Esophageal squamous cell carcinoma
FP: Fluorescence polarization
GBM: Glioblastoma
GC: Gastric cancer
GCB: Germinalcenter B cell-like
HCC: Hepatocellular carcinoma
HIF-1: Hypoxia-inducible factor 1
HIF1A: Hypoxia-inducible factor 1A
HNSCC: Head and neck squamous cell carcinoma
HTRF: Homogeneous time resolved fluorescence
ICC: Intrahepatic cholangiocarcinoma
IR: Ionizing radiation
LFT: Liver function test
LSC: Leukemia stem cells
MAPK: Mitogen-activated protein kinase
miR-155: Microrna-155
MM: Multiple myeloma
MMP9: Matrix metalloproteinase 9
mTOR: Mammalian target of rapamycin
MW: Molecular weight
NAE: NEDD8-activating enzyme E1
NEDD8: Neuronal precursor cell-expressed developmentally downregulated protein 8
NF-κB: Nuclear factor-κB
NPC: Nasopharyngeal carcinoma
NSCLC: Non-small cell lung cancer
OCC: Ovarian cancer cell
PCa: Prostate cancer
PDL1: Programmed death ligand-1
PDTX: Patient-derived tumor xenografts
PK: Pharmacokinetics
RBX1: RING-box protein 1
RBX2: RING-box protein 2
ROS: Reactive oxygen species
SAE: SUMO-activating enzyme
SARs: Structure-activity relationships
SCC: Squamous cell carcinoma
SCF: Skp1-Cul1-F-box protein
SI-NETs: Small intestinal neuroendocrine tumors
Skp1: S-phase kinase associated protein 1
TAMs: Tumor associated macrophages
TCGA: Cancer Genome Atlas database
TGFβ: Transforming growth factor
TNF-α: Tumor necrosis factor-alpha
TR-FRET: Time-resolved fluorescence resonance energy transfer
UAE: Ubiquitin activating enzyme
UBA3: Ubiquitin-like modifier activating enzyme 3
UBC12: NEDD8 conjugating enzyme
UBE2F: Ubiquitin-conjugating enzyme E2F
UC: Urothelial carcinoma
UM: Uveal melanoma
UPS: Ubiquitin-proteasome system
α-SMA: Α-smooth muscle actin
